# Overactivation of SHH signaling induces a cascade of dedifferentiation and oncogenesis in a new mouse model for choroid plexus carcinoma

**DOI:** 10.1186/s40478-026-02402-y

**Published:** 2026-08-21

**Authors:** Levke-Sophie Peter, Lea Altendorf, Karoline Hack, Vanessa Thaden, Carolin Göbel, Beatrix Mahnke, Christian Krebs, Saskia-L. Jauch-Speer, Uwe R. Kordes, Denise Obrecht-Sturm, Christian Thomas, Ulrich Schüller, Melanie Schoof

**Affiliations:** 1 Research Institute Children’s Cancer Center Hamburg, University Medical Center Hamburg-Eppendorf, Martinistraße 52, Building N63, 20251 Hamburg, Germany; 2 Department of Pediatric Hematology and Oncology, University Medical Center Hamburg-Eppendorf, Hamburg, Germany; 3 III. Department of Medicine, University Medical Center Hamburg-Eppendorf, Hamburg, Germany; 4 Hamburg Center for Translational Immunology, University Medical Center Hamburg-Eppendorf, Hamburg, Germany; 5 Single Cell Core Facility, University Medical Center Hamburg-Eppendorf, Hamburg, Germany; 6 Institute of Neuropathology, University Hospital Münster, Münster, Germany; 7 Institute of Neuropathology, University Medical Center Hamburg-Eppendorf, Hamburg, Germany; 8 Mildred Scheel Cancer Career Centre HaTriCS4, University Medical Centre Hamburg-Eppendorf, 20246 Hamburg, Germany

**Keywords:** Choroid Plexus carcinoma, Sonic Hedgehog signaling, Dedifferentiation, Spatial transcriptomics, Mouse model

## Abstract

Choroid Plexus tumors constitute 10–20% of brain tumors in infancy. Among these, Choroid Plexus Carcinomas (CPC) are highly aggressive and result in poor survival. Details on tumor initiation and oncogenic events remain largely unknown, aside from a high prevalence of *TP53* mutations and marked chromosomal instability. We generated *hGFAP-cre::lsl-MYCN::lsl-Gli2(N)::Tp53*^*fl/fl*^ mice with recombination activity from embryonic day 13.5 onwards in CNS cells, leading to *MYCN* and *Gli2* activation as well as *TP53* inactivation. 84% of the mice developed ventricular tumors within 18 days of life, resembling human CPCs in histology and marker expression. We performed histological and molecular characterization of the resulting tumors and treated tumor cells in vitro. We demonstrate that aberrant Sonic Hedgehog (SHH) signaling during embryonic Choroid Plexus (CP) development triggers a dedifferentiation cascade that reverts mature epithelium to a progenitor-like state. Spatial transcriptomic analysis identifies a distinct cellular hierarchy, where differentiated plexus cells shed their secretory identity to adopt malignant progenitor states. This transformation is accompanied by a transition from multiciliated epithelial cells expressing mature, secretory CP markers to highly proliferative, monociliated progenitors expressing SOX2. This malignant reprogramming activates embryonic developmental programs and induces a critical dependency on cell cycle and DNA repair pathways. Consequently, we identify that these tumor cells cultivated *in vitro* are vulnerable to ATR and CDK inhibition.

**Supplementary Information:**

The online version contains supplementary material available at 10.1186/s40478-026-02402-y.

## Introduction

Choroid Plexus Tumors (CPT) are rare pediatric neoplasms comprising 10–20% of brain tumors in the first year of life [5, 52]. CPTs are classified into three histological subtypes, Choroid Plexus Papilloma (CPP), atypical Choroid Plexus Papilloma (aCPP), and Choroid Plexus Carcinoma (CPC) [36]. The latter are particularly aggressive and between 20 and 60% of patients present with metastasis at initial diagnosis [35, 69]. Children typically undergo surgical resection with adjuvant chemotherapy or radio-chemotherapy [28, 69]. Despite this aggressive therapy, 5-year progression free survival is still only 50% [5, 63, 69]. Additionally, no targeted treatments exist and surviving patients often suffer severe long-term effects following treatment [69]. Previous research highlights *TP53* mutations as a recurring aberration in about 50% of overall cases [62, 65], supported also by the development of CPCs in Li-Fraumeni syndrome patients with germline *TP53* mutations [48]. Otherwise, CPCs remain molecularly incompletely understood, exhibiting diverse large-scale chromosomal aberrations resulting in a generally hypodiploid chromosomal state. Global DNA methylation profiling reliably differentiates CPCs into three methylation groups [7, 41, 49, 64]. All CPCs cluster into one epigenetic high-risk subgroup (“pedB”).

In order to gain more knowledge on the molecular mechanisms of CPT development, mouse models have been previously developed [14, 42, 58]. Research so far has revealed that combining *c-Myc* overexpression with *TP53* loss drives CPT formation [68]. Additionally, the combination of *TP53*, *Rb* and *Pten* loss resulted in CPC formation in mice, leading to the identification of three potential oncogenes *Taf12*, *Nfyc,* and *RAD54L* [66]. However, all of the existing models are limited by either nonviability or a relatively late tumor onset and long survival until postnatal day (P) P150-P200. Other pathways like SHH and Notch signaling have also been implicated in tumor development [33], mainly because of the embryological and developmental background of choroid plexus (CP) tissue. During development, hindbrain CP is formed by sequential contributions of progenitor pools formed from lateral roof plate and lower rhombic lip cells, during which rhombomeric spatial distribution is preserved [2, 21, 30, 37, 50]. SHH, Wnt, and Notch signaling are essential for hindbrain plexus development and especially important for the formation of ciliary structures, which contribute to the elemental function of CP: production and circulation of cerebral spinal fluid (CSF) [20, 45]. *Gli2* is part of the GLI family and is a key downstream target in SHH signaling and thus acts as transcriptional effector. It is known to be frequently amplified and overexpressed in pediatric brain cancer. *MYCN* is a transcription factor belonging to the MYC family. *MYCN* is expressed in neural tissues and essential for normal central nervous system (CNS) development [8, 29, 53]. SHH signaling is crucial for CP development, *MYCN* is a potent oncogene in neural tissues, and *TP53* is the most frequently mutated gene in human CPCs. We therefore hypothesized that the concurrent activation of SHH signaling, through the combination of *Gli2* and *MYCN* overexpression and the *TP53* loss, would be sufficient to drive CPC formation. Our resulting new mouse model provides a new platform for the study of this lethal disease.

## Methods

### Ethics

All work in this project complies with relevant ethical regulations, including Hamburg’s animal protection laws and the Institutional Animal Care and Use Committee guidelines from the University Medical Center Hamburg‑Eppendorf (§12 HmbKHG). Only anonymized human data were included in the project.

### Transgenic mice

The generation of *hGFAP-cre* [77], *Tp53*^*fl/fl*^ [25], *CAG-lsl-Gli2* [46]*, lsl-MYCN* [1] and *hGFAP-cre::Tp53*^*fl/fl*^*-lsl-MYCN* [56] mouse lines has been described previously. All animal procedures were performed per applicable animal protection laws and approved by the state of Hamburg (Reference N2019/99). Mice were housed at 22 °C, 45–65% humidity, on a 12‑h light/dark cycle with food and water ad libitum. Mice were monitored daily and euthanized upon onset of neurological symptoms (ataxia, hydrocephalus). No termination criteria were exceeded. Genomic DNA from tail tips was used for genotyping via standard DNA extraction, gel electrophoresis and PCR with primers listed in Supplementary Table 1.

### Primary murine tumor cell lines

Fresh hindbrains from three mice were dissected and dissociated with Accutase and DNase. Cells were seeded in NSC medium (DMEM/F12 + Glutamax, HEPES, NEAAs, P/S, B27, EGF, FGF) and incubated at 37 °C. Once spheres formed, they were passed and dissociated. EGF and FGF were refreshed every 2–3 days. Tumor cell features were confirmed by centrifuging cell pellets, fixing with 4% paraformaldehyde/PBS for ≥ 12 h at 4 °C, dehydrating, embedding, and staining at standard protocols. Control untransformed NSC cells were generated from forebrain samples of E12-E14 old embryos with a C57BL/6 J or lsl-R26tfRFP background as previously described [3]. In short, a pregnant mouse was sacrificed, embryos were placed in Hanks Balanced Salt Solution, tissue was dissociated using Accutase and a cellstrainer, and cells were cultivated as described above.

### Differentiation assay

Differentiation assays were performed as previously described [55]. Briefly: To facilitate cell adhesion, coverslips were coated with poly-L-ornithine (#P3655-100MG, Sigma) overnight in a freezer, washed with PBS and coated with laminin (#L2020-1MG, Sigma Aldrich) for 1 h at 37 °C beforehand. Subsequently, cells were cultured in medium containing 10% fetal calf serum (FCS) (#10,270,106, Life Technol.) to induce differentiation. After 7 days in culture, cells were fixed by PFA (10 min). Immunofluorescence staining was performed, and 10 representative pictures of every coverslip were taken and analyzed using NIS-Elements AR software (5.11.03).

### Immunofluorescence assay

For immunofluorescence staining, blocking used 10% normal goat serum (NGS) (#S26-100ML, Merck Millipore) and staining with primary antibodies: anti-GFAP (#14-9892, Invitrogen, mouse IgG1, 1:500), anti-TUBB3 (#T2200, Sigma, rabbit IgG, 1:200), anti-Ki67 (#ab16667, RRID:AB_302459, Abcam, rabbit, 1:200), anti-OLIG2 (#AB9610, RRID:AB_570666, Millipore Sigma, rabbit IgG, 1:500), anti-SOX2 (#Mab2018, RRID:AB_358009, R&D systems mouse IgG, 1:200). Fluorescence detection used species-specific fluorophore linked secondary antibodies: Alexa 555 α-mouse (#CST4409, RRID:AB_2943224, Cell Signaling, 1:500) and Alexa fluor 488 α-rabbit (#CST4412, Cell Signaling, 1:500). DAPI (#28718-90-3, Roth, 1:1000) stained cell nuclei. A microscope (Eclipse Ti2-E, Nikon) was used for analyses.

### Drug testing

Cells were seeded at 10^3^ per well in 100 µl NSC medium, grown 24 h, then treated with six inhibitor doses (10 µl). Untreated and DMSO‑treated wells served as controls. BKM-120 (#HY-7006, MedChem Express) and Dinaciclib (#S2768, Selleckchem) stock solution were 1 mM in 100% DMSO. Tuvusertib was generously provided by Nina Struve as a 4 mM stock solution in 100% DMSO. Fresh solution was prepared prior to testing using PBS and NSC medium. Following 24 h inhibitor treatment, medium was replenished with 50 µl. Viability was measured 5 days post‑seeding using CellTiter‑Glo (Promega) and a Spark plate reader (Tecan). Inhibitor-dose–response curves and AUC values were generated using GraphPad Prism (v9.5.0). All experiments were conducted using three different tumor derived cell lines, three biological replicates and three technical replicates per biological replicate. Control cell lines from two different non-transformed mouse cell lines (see above) were included with three technical replicates each. AUC comparison was visualized using R and reference data from Genomics of Drug Sensitivity in Cancer Release 8.5 [71]. All AUC and IC50 values are in Supplementary Table 2.

### Histology and Immunohistochemistry

For hematoxylin and eosin (H&E) and immunohistochemistry (IHC) stains, brain tissue was fixed (4% paraformaldehyde/PBS, > 12 h). The tissue was dehydrated, embedded in paraffin, and sectioned at 3–5 μm according to standard protocols. All stains were performed on a Ventana System (Roche) using standard protocols. Antibodies used are: Arl13b (Proteintech, 17,711–1-AP, RRID:AB_2060867, 1:200), AQP1 (Merck, AB2219, 1:200), Doublecortin/DCX (Abcam, ab18723, RRID:AB_732011, 1:100), E-Cadherin (Dako IR059, RRID:AB_2341210, ready to use), Foxj1 (Sigma, HPA005714, RRID:AB_1078902, 1:100), Ki67 (Abcam, ab16667, RRID:AB_302459, 1:100), MYCN (Cell Signaling, 517,053, 1:1000), NeuN (Millipore, MAB377, RRID:AB_2298772, 1:25), Nestin (Abcam, ab221660, RRID:AB_2909415, 1:2000), SOX2 (Abcam, ab97959, RRID:AB_2341193, 1:200), OLIG2 (Millipore, AB9610, RRID:AB_570666, 1:200), OTX2 (Thermo Scientific, 1H12C4B5, 1:250), RFP (Antibodies Online, ABIN129578, RRID:AB_10781500, 1:75), Transthyretin (ThermoFischer, PA5-88,094, RRID:AB_2804646, 1:50), Kir7.1 (kindly provided by Christian Thomas, 1:2000).

### Chromatin density quantification

Representative H&E images from control (n = 6) and mutant (n = 6) mice were processed in ImageJ (RRID:SCR_003070). Color deconvolution separated channels; cells were segmented and thresholded (blue ≥ 170) to classify chromatin. Percent of normal vs. abnormal nuclei was calculated automatically.

### Transcriptomic data generation

We sequenced 6 tumor samples from 4 mice (matched forebrain/hindbrain pairs at P14, P15, P17; 1 male, 3 female). Samples were prepared using the 10X Visium CytAssist for FFPE protocol, including hydration, cooling, slicing (5 µm), deparaffinization, staining, and decrosslinking according to 10X guidelines (CG000518–CG000521). Samples were imaged using a BX43F microscope and hybridized using whole-transcriptome probes. Probes were ligated, extended and transferred to barcoded capture areas via CytAssist (CG000495). Libraries were pooled and sequenced on a NovaSeq 6000 (Cegat) with 182.193–494.318 Mio. reads/sample. Read quality was confirmed by FastQC (RRID:SCR_014583) version 0.11.5-cegat49 (Babraham Bioinformatics—FastQC A Quality Control tool for High Throughput Sequence Data). Further quality control is available in Supplementary Table 3.

### RNA sequencing analysis

Fastq files were processed in Space Ranger v3.1.0. (RRID:SCR_025848), including low quality trimming, barcode correction, probe filtering, and UMI counting. Reads were aligned to refdata-gex-mm10-2020-A.tar.gz. 10X Output was analyzed in R using Seurat (RRID:SCR_016341, v 5.1.0.) [17]. Samples were filtered (low ncounts), normalized (SCTtransform), and integrated with harmony (v1.2.1) [31]. Feature plots, UMAPs [40], dot plots, and heatmap visualizations were created with Seurat. Annotation used scType (github commit 6db9eef) with published datasets as reference [59, 75] (see also Figure S3). Subcallosal zone scoring was based on differential gene expression from reference data [27], with a 0.3 cut-off. Visualizations used ggplot2 (RRID:SCR_014601, v 3.5.1) and ggrepel (v 0.9.5). Differential expression was calculated with Seurat FindMarkers (*p*-value < 0.05 and log2fold change > 0.6). Statistical significance was calculated using a Wilcoxon Rank Sum test, and *p*-values were adjusted for multiple testing using the False Discovery Rate (FDR) correction (Bonferroni). Results with an adjusted *p*-value < 0.05 were considered statistically significant. Spot deconvolution was performed using Seurat. GSEA analysis, generation of KEGG pathways (RRID:SCR_012773) and diagrams [26, 38] used ShinyGo v 0.81 [15]. Trajectory analysis used monocle3 (v 1.3.7) [6, 51, 67]. Human data were downloaded from Gene Expression Omnibus (GEO) database GSE264154 [19] and analyzed using Seurat [17]. Copy number profiling was performed on the merged raw gene expression counts matrix with methods for bulk RNA sequencing data using InferCNV of the Trinity CTAT Project (https://github.com/broadinstitute/inferCNV). Public methylation data of n = 28 CPC tumors in the pedB methylation class from GEO database GSE109381 [7] were analyzed using DIMEImmune [54]. Signaling interaction was computed using Cell Chat (v 2.2) [22] integrating gene expression with spatial data and thus inferring intercellular communication. Analysis was performed independently on Samples A, B and C.

### Cross-species analysis

To compare murine tumors with different human tumor entities, a cross-species analysis was performed in R Studio (v4.4.1). Gene expression array data [61] and bulk RNA-seq data [23, 65] covering various CNS-tumors were used as human references. To validate this analysis RNA-seq data from MB-SHH developing *Math1-cre::Smo*^*fl/wt*^ mice were [57] included. Each dataset was separately VST-normalized using DESeq2 (RRID:SCR_000154, v1.46.0) and Z-score-normalized. Count tables were merged and batch effects from species or platforms were corrected using limma (RRID:SCR_010943, v3.62.2). The 1,000 most variable genes among all samples were calculated. Results were visualized in a UMAP (RRID:SCR_018217, v0.2.10.0) with 10 neighbors, and a heatmap, using the ComplexHeatmap package (RRID:SCR_017270, v2.22.0). The clustering distance metric was defined as “pearson” and the method was set as “average”. RNA microarray data [66] were used to compare different mouse models.

### Statistics

Kaplan–Meier (KM) curve, chromatin density bar plot and drug-response curves were generated using GraphPad Prism 9 (RRID:SCR_002798) software. KM-curve included animals sacrificed due to occurring symptoms with histologically visible tumor. Error bars represent the standard error of mean. Significant p-values and sample sizes (n) are reported in the figure legend. Penetrance was calculated using the total number of mice with appropriate genotype aged P5 or older and available histology (n = 50) and visible tumor development (n = 42).

### Data availability

All transcriptomic data are available under the GEO Accession number GSE289052.

## Results

### Alteration of Tp53, MycN, and Gli2 drive CPC formation in mice

**Fig. 1:**
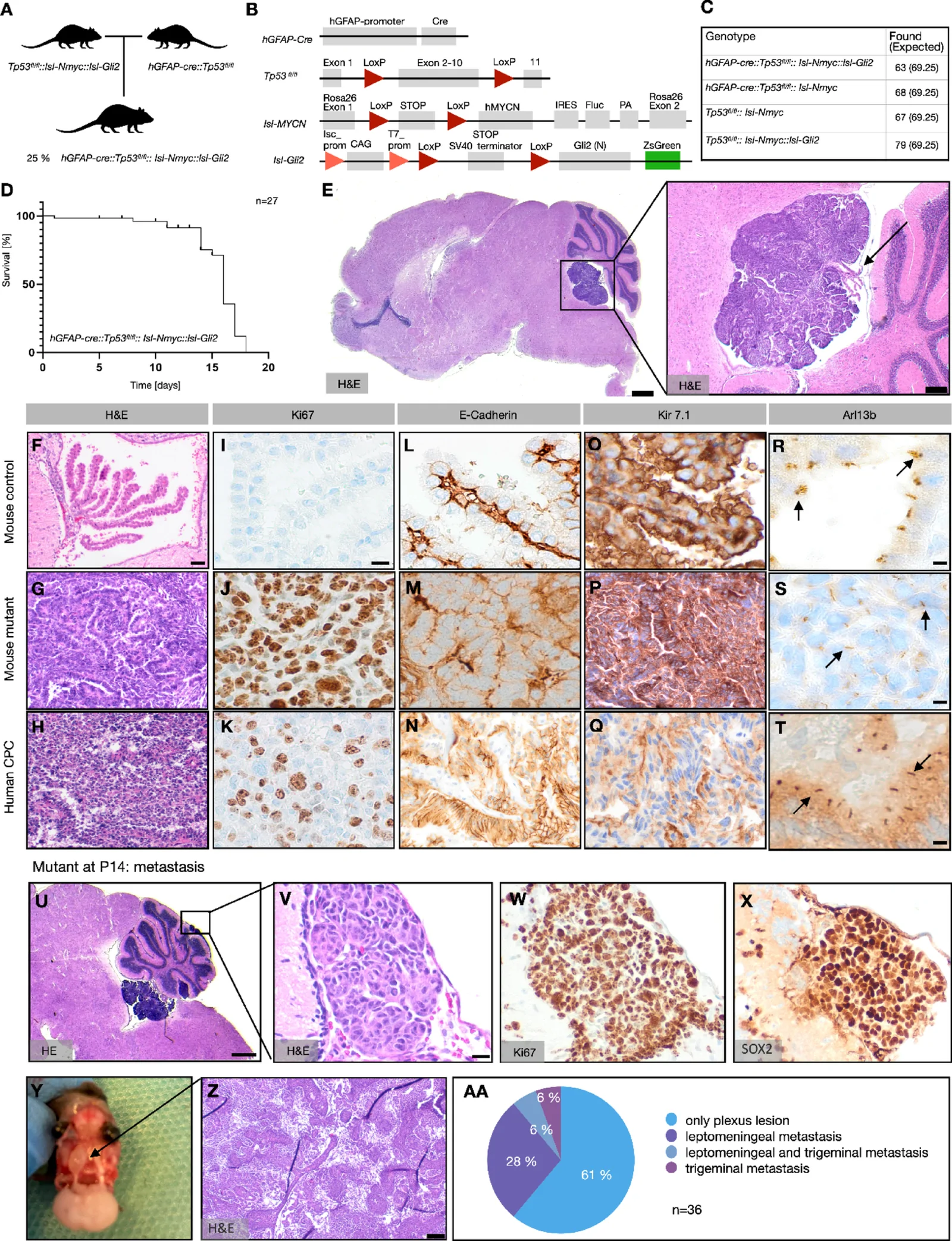
*Tp53*, *Gli2* and *MYCN* altered mice develop tumors within 18 days and show histological similarity to human CPCs. **A** Breeding scheme of *hGFAP-cre::Tp53*^*fl/fl*^*:: lsl-MYCN::lsl-Gli2* mice. **B** Genetic alterations introduced in the GEMM, Cre is expressed under control of the *hGFAP* promoter. The floxed alleles of *Tp53, MYCN,* and Gli2 genes are depicted, loxP sites are shown as red arrows, reporter fluorescence genes as green boxes. **C** Genotype frequency observed in the mouse model as expected by Mendel’s law. **D** Kaplan–Meier survival curve showing survival of n = 27 mice. **E** H&E stain showing sagittal section of a P14 mouse brain with visible hindbrain tumor (inset), arrow marks 4th ventricle hindbrain CP tissue. **F** Histological comparison between mouse control, mouse tumor and human CPC sample revealing comparable histology and marker expression. Ciliary staining in fourth column, arrows are marking multiciliary healthy plexus cells in the top row and monociliary tumor cells in mouse and human tumors below. **G** H&E stain of a P14 mouse with leptomeningeal drop metastasis shown with histological characterization of metastasis. **H** Macroscopically visible trigeminal lesion shown in H&E staining in (**I**), displaying similar architecture to hindbrain lesions. **J** Pie chart with occurrence of metastatic disease n = 36. All histological analysis was confirmed by at least three independent samples

To establish a mouse model for human CPC we employed the Cre-LoxP system under the human glial fibrillary acidic protein (*hGFAP)* promoter targeting radial glia cells from as early as embryonal day (E) 13.5 onward. *Tp53*^*fl/fl*^*::lsl-MYCN::lsl-Gli2* mice were crossbred with *hGFAP-cre::Tp53*^*fl/fl*^ mice (Fig. 1A). A loss of p53 by loxP-directed recombination after exon 1 and 10 in the *Tp53* gene is combined with the overexpression of human *MYCN* gene and addition of mutated *Gli2A* (Fig. 1B). The resulting genotypes matched the expectation, *hGFAP-cre::Tp53*^*fl/fl*^*::lsl-MYCN::lsl-Gli2* (hereafter called mutant) mice were born with a frequency of 22.7% (25% expected) indicating no significant intrauterine death (Fig. 1C). Observed mice with this genotype became symptomatic within the first 18 days of life. Most developed visible hydrocephalus, ataxia and imbalance and were then sacrificed. Survival analysis indicates early tumor development with symptomatic cases as early as at P8 (Fig. 1D). Of the 50 mice older than P5 included we could detect tumor cells in 42, a penetrance of 84%. Mutant mice were examined for possible tumor development and had visible hindbrain tumors all localized to the fourth ventricle. The cerebellar structure was identical to control littermates, and no other cerebellar or hindbrain malformations were observed. Tumors developed in anatomical continuity to visible, regularly configured fourth ventricle CP tissue (Fig. 1E). A detailed analysis of tumor histology was performed revealing disturbed morphology compared to the characteristic classical single-layer cobblestone-like appearance of healthy CP tissue (Fig. 1F). Mutant mice presented a cell-dense tumor with visibly altered nuclei and an overall loss of architectural organization, barely resembling normal CP organization. The tumor cell morphology appears similar to human CPC (Fig. 1F). These architectural changes align with the 2021 WHO Classification of Tumors of the Central Nervous System histological criteria [36], fulfilling four of the five criteria: blurring of the papillary pattern, nuclear pleomorphisms, increased mitoses, and cellular density but missing necrosis typically present in late human tumor stages. Aggressive growth and high proliferative index were confirmed by increased Ki67 staining in both mouse and human tumor samples (Fig. 1F). Since CP is an epithelial tissue, markers such as E-Cadherin further support CPC diagnosis in humans, and respective expression can also be seen in mice. Another key diagnostic marker, the epithelial inward rectifier channel Kir 7.1, was also expressed (Fig. 1F). Additional histological characteristics include Cytokeratin expression and negativity for neural markers (Fig. S1). CP precursor cells are monociliated and differentiate into mature, multiciliary cells in the adult CP tissue [20] that facilitate CSF flow by their beat. This multiciliation is lost in mouse and human CPC tumor cells, as shown by Arl13b-stained single cilia (arrows, Fig. 1F). This single cilium represents the SHH signaling hub during CP development [13, 44], suggesting a process of dedifferentiation into a signaling state similar to that of undifferentiated precursor cells.

CPC tumors in humans are aggressive malignancies associated with early onset, rapid progression and a high rate of metastases, most often as leptomeningeal drop metastases [47]. Mouse tumors exhibited a similar tumor progression and metastatic pattern, with cerebellar meningeal metastasis sharing a close histological resemblance and similar marker expression to the 4th ventricle tumors (Fig. 1G). Macroscopically visible metastases at the trigeminal nerve were observed in 12% of mice (Fig. 1H–J and Fig. S2).

### Tumors arise from a stepwise dedifferentiation which matches embryonic processes

**Fig. 2:**
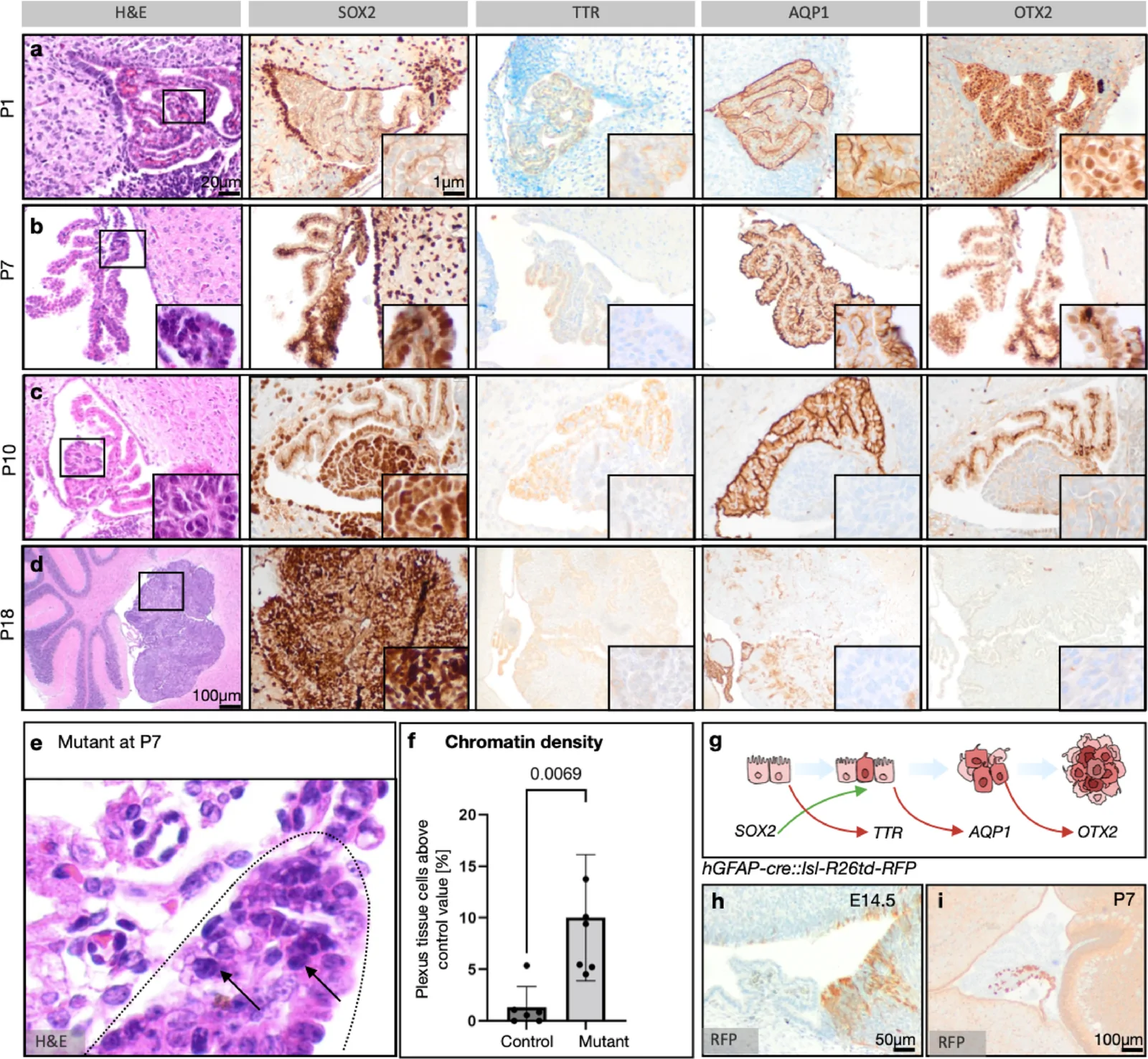
Tumor development reveals a stepwise dedifferentiation process. **A**–**D** Characterization of tumor development of *hGFAP-cre::Tp53*^*fl/fl*^*::lsl-Nmyc::lsl-Gli2* mice, P1 Plexus (**A**) displaying normal marker expression, H&E stain from mutant mice at P7 (**B**) displaying noticeable changes in cell architecture (inset), showing increased SOX2 positivity, loss of TTR expression, and retained AQP1 and OTX2 expression. **C** Further lesion development in P10 mice showing larger aberrant structures. The marker comparison is revealing further loss of AQP1 and OTX2. **D** Final marker profile of P18 mice with large lesions. All analysis was performed on at least three different animals with lesions of similar size. **E** H&E stain of beginning lesion in P7 mouse, arrows: aberrantly dark chromatin. **F** Bar plot visualizing chromatin quantification, percent cells above chromatin density threshold were measured for n = 6 per group, *p* = 0.0069 significance threshold *p* < 0.05, independent t-test. **G** Schematic summary of tumor progression, red arrows indicating marker loss, green arrows indicating higher expression, cells with darker and larger nuclei and monocilliation are depicted as the tumor progresses. **H** Lineage tracing with *hGFAP-cre::lsl-R26td-RFP* mice showing no RFP expression in embryonal day E14.5 mouse and **I** showing specific expression pattern in P7 mouse, corresponding to differently contributing rhombomeric precursor populations. hGFAP expression pattern was confirmed using at least three samples and RFP staining in adult mice at P144

Tumor development was further characterized by observing mutant mice at various time points. At P1, CP architecture showed no evident changes, with physiological expression of SOX2, TTR, AQP1, and OTX2 expression as reported in the literature (Fig. 2A) [9, 24]. Early signs of tumor development are visible at P7 and include nuclear changes with increased pleiomorphisms and an aberrant cell morphology (Fig. 2B). These changes coincided with increased SOX2 expression and loss of the plexus marker TTR, while AQP1 and OTX2 expression was retained. Further progress of tumor growth is visible at P10, where the architectural organization in cobblestone patterning and row structures of the CP tissue are lost and replaced by a nodular growth (Fig. 2C). At this stage, tumor cells also lost AQP1 and OTX2 expression. Large tumors at P18 (Fig. 2D) continue to display this marker pattern. Changes in these characteristics were linked to tumor growth stages rather than mouse age; for example, small lesions at P11 resembled P7 tumors displayed here. Nuclear structure changes, including higher chromatin density compared to control CP nuclei, were early indicators of tumor lesions (Fig. 2E, F).

The development of murine CPTs follows a reproduceable pattern of differently expressed markers by dedifferentiation of multiciliated, healthy CP cells gaining SOX2 expression and losing TTR expression as well as transitioning to a monociliary state with increased chromatin density. Tumor progression then also involves the stepwise loss of AQP1 and OTX2 (Fig. 2G). These markers reflect a dedifferentiation process and acquisition of a precursor-like malignant state. Tumor cells thus resemble CP precursor cells and their embryological signature where SHH signaling plays an especially important role in the development of the hindbrain plexus.

**Fig. 3:**
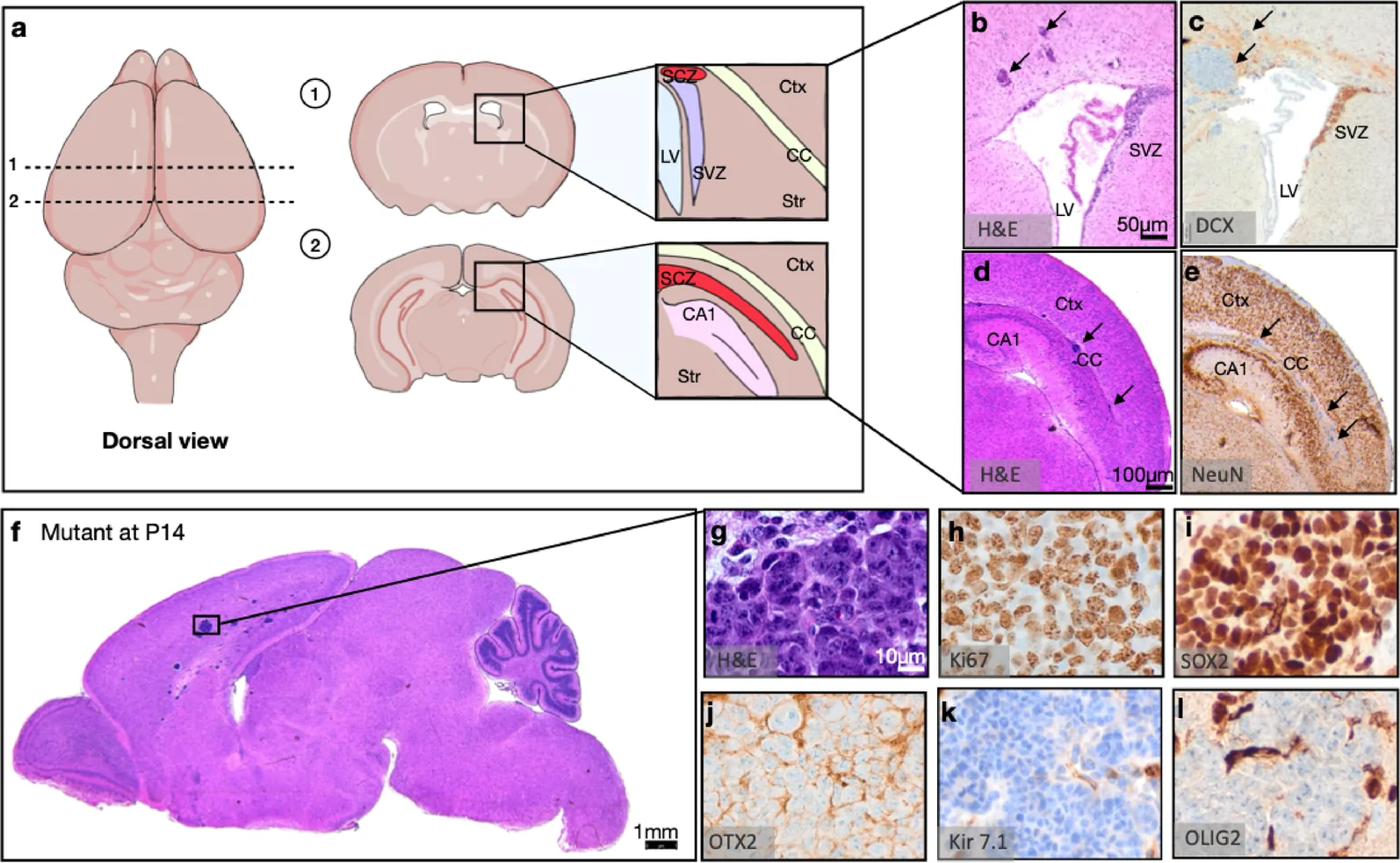
Mutant mice also develop forebrain lesions localized to the subcallosal zone. **A** Schematic view of dorsal mouse brain indicating two sectional planes with schematic annotation of brain areas, LV = lateral ventricle, SCZ = subcallosal zone, SVZ = subventricular zone, Str = Striatum, CC = Corpus callosum, Ctx = Cortex, CA1 = hippocampal subfield (cornu ammonis 1). **B**, **C** Anatomical position of the forebrain lesion in relation to LV and SVZ, arrows indicating forebrain lesion, doublecortin (DCX) staining marking SVZ neural progenitors. **D**, **E** Forebrain lesion position relative to hippocampus and corpus callosum visualized though neuron free section, lesions lie above hippocampal structures and below cortical neuronal layers. **F** H&E stain of P14 mutant mouse with forebrain lesions arranged in a chain like fashion. **G** H&E inset of forebrain lesion architecture, the tumors are highly proliferative (**H**), express SOX2 (**I**) but lack expression of OTX2 (**J**), Kir7.1 (**K**) and OLIG2 (**L**)

In humans, CPCs predominantly arise in the lateral ventricles [64]. In children, CPCs rarely occur in the 4th ventricle. In the analysis of Meyer et al. 1 out of 11 CPCs in pediatric patients presented there [43]. In adults, CPCs occur more often in the 4th ventricle [70]. However, all mouse lesions consistently arise from one cell population at the anatomical border of ependymal cells and CP tissue in the 4th ventricle. To investigate this appearance pattern, we performed lineage tracing with *hGFAP-cre::lsl-R26td-RFP* mice (Fig. 2H, I). We found that *hGFAP* targets a specific portion of the hindbrain plexus, not the lateral ventricles, potentially reflecting the sequential development of CP tissue. Sequential contributions from different precursor cells create a spatial and temporal pattern in CP development. The cells targeted here correspond to cells derived from rhombomeres 2–8. The dedifferentiation and tumor formation processes are dependent on the specific environment of the CP. The same oncogenic driver combination also leads to the formation of multiple forebrain lesions with a consistent chain-like arrangement. We examined different levels and orientations to further identify the affected region (Fig. 3A). The lesions lie separate from the subventricular zone (SVZ) (Fig. 3B, SVZ clearly visible by a positive DCX staining in Fig. 3C) and above the lateral ventricles as well as the hippocampal structures (Fig. 3D, E). This localization is most consistent with the subcallosal zone (SCZ), which is characterized by the existence of a persistent neural progenitor pool similar to the SVZ. The SVZ and SCZ are two distinct regions with different transcriptional profiles, and the neural SCZ progenitors are subject to *MYCN* signaling, providing a rational for tumor development in this model. The lesions are made up of densely packed cells (Fig. 3F, G) and are highly proliferative in Ki67 staining (Fig. 3H). Even though they share the SOX2 expression (Fig. 3I) and are negative for OTX2 (Fig. 3J), similar to the hindbrain lesions, they do not express Kir 7.1 (Fig. 3I). Since those forebrain lesions arise simultaneously with the hindbrain lesions and show distinct histological features, they represent an independent undifferentiated tumor with a presumed neural progenitor origin in the SCZ. This is also supported by their neurospheric growth in cell culture and their ability to differentiate into multiple lineages (Fig. S3).

### Transcriptomic signature of tumor cells matches embryological ChP and human CPCs

**Fig. 4:**
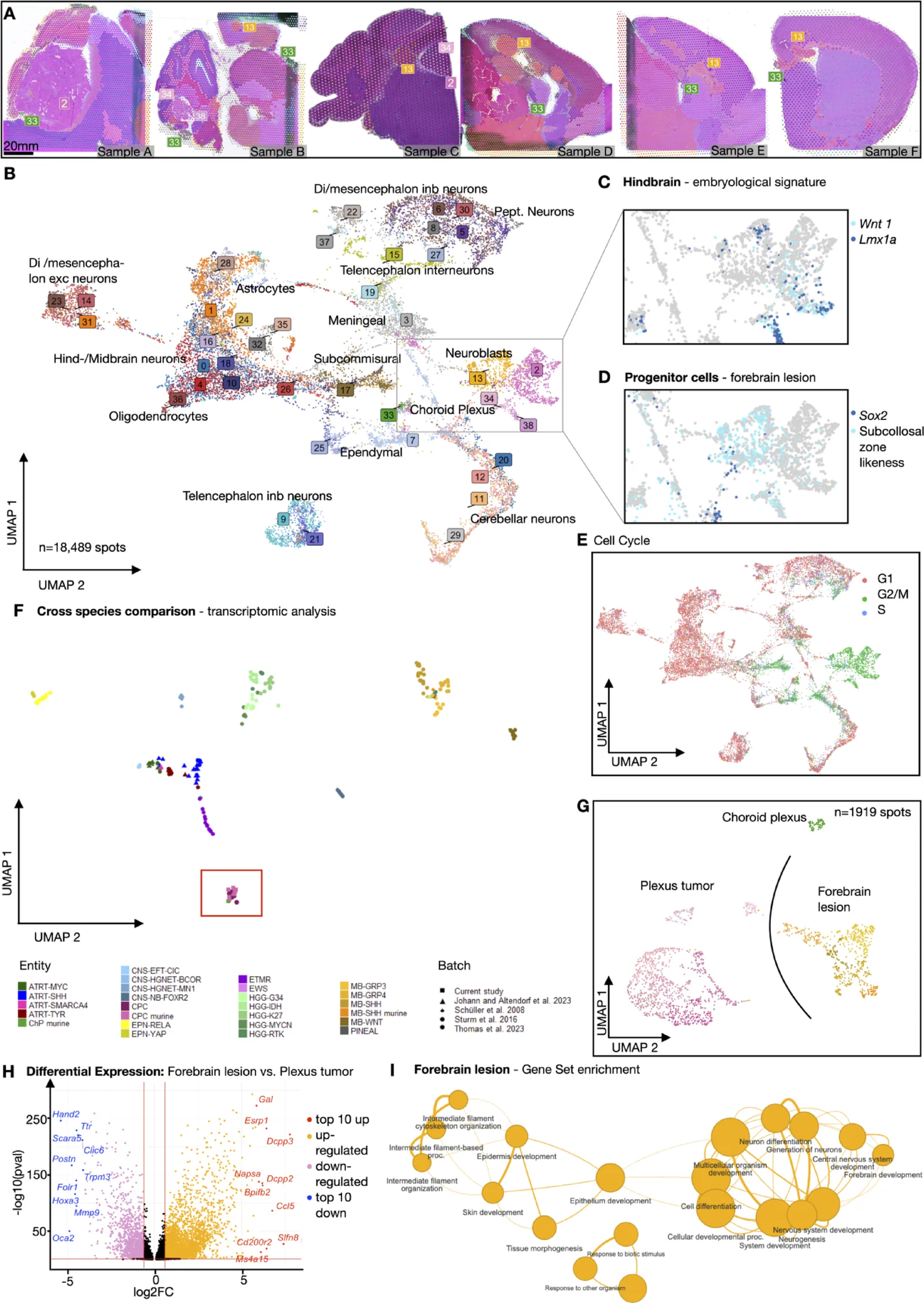
Spatial transcriptomic analysis of hindbrain and forebrain lesions show CPT and SCZ neural progenitor signature respectively. **A** H&E stained slides from Samples A-F used for RNA sequencing, spots are mapped onto histological background using Space Ranger and colored according to Seurat-clusters. Clusters 2, 34, and 38 (pink colors) represent hindbrain tumors, Cluster 33 (green) can be identified as healthy plexus, even if only 1–2 spots cover the area such as in sample E) or F), Cluster 13 (yellow) consists of forebrain lesions. **B** Uniform Manifold Approximation and Projection (UMAP) of n = 18,489 spots integrated across all samples and annotated according to a reference atlas; numbers indicate Seurat clusters. Cells in cluster 13 are annotated as neuroblasts, clusters 2, 34, and 38 are annotated as choroid plexus but cluster distinctly from cluster 33, which is annotated as choroid plexus as well. **C**, **D** Subset of UMAP in **B** showing Wnt1 and Lmx1a expression in CP tumor clusters usually only expressed in embryological CP tissue (**C**) and expression of Subcallosal zone likeness score as well as Sox2 expression in forebrain tumors (**D**). **E** Cell cycle scoring applied to UMAP in **G** showing highly proliferative tumor cells from both lesions mostly in G2/M phase (green). **F** Transcriptomic comparison to human reference data of CNS neoplasms using the 1000 most variable overlapping genes. Mouse samples CPC_murine (pink) match reference human data CPC (violet). As a control, mouse SHH medulloblastoma (MB-SHH murine, orange) were included and perfectly match to human MB-SHH (gold). **G** Reclustered UMAP of subsetted clusters 13, 33, 34, 38, and 2 showing plexus and forebrain tumors clustering distinctly, different colors correspond to subgroups of different samples A-E, n = 1919 spots. **H** Volcano plot of DEG from forebrain and plexus tumors highlighting differences in gene expression. **I** Gene set enrichment analysis of forebrain tumors predominantly showing neuronal development pathways. Size of the circles corresponds to number of genes in pathway, nodes are connected if more than 20% genes overlap, thicker edges represent more overlapping genes

To better understand the molecular characteristics of the tumors we performed spatial transcriptomics using the 10X Visium HD platform. We analyzed six samples from three primary plexus tumors and three primary forebrain lesions from four mice (Fig. 4A) overall resulting in n = 18,489 data spots, each comprising 2–5 cells. Data integration and clustering identified 38 distinct clusters (Fig. 4B), including four distinct tumor cell populations (2, 34, 38, and 13). We annotated clusters using a reference atlas [59] and validated them with spatial sample information (Fig. S4, annotation data can be found in Supplementary Table 4). Hindbrain lesion cells (2, 34, and 38, pink) mostly resemble CP tissue but cluster distinctly from morphologically normal appearing CP tissue (33, green). Forebrain lesion cells (yellow, 13) most closely resemble neuroblasts, supporting our hypothesis of neural progenitor cells as the potential cell of origin. Plexus lesion cells express signature markers usually only present in embryological CP tissue such as *Lmx1a* and *Wnt1* which both play an essential role in CP development (Fig. 4C) [10].

To investigate the forebrain lesion’s origin, we calculated a subcallosal zone likeness score using SCZ gene expression reference data (Fig. 4D) [27]. Cell cycle scoring confirmed the tumors’ high proliferative nature, with most cells in the G2/M phase (Fig. 4E).

In order to further validate our model, we mapped our tumors to a broad reference atlas of human CNS neoplasms. The expression pattern of our samples most closely matches the signature of human CPCs and a strong similarity of expression pattern is observed (Fig. 4F).

Reclustering hindbrain and forebrain lesion cells with CP tissue confirmed their distinct nature, with minimal overlap between lesions (Fig. 4G). To further characterize the difference of these tumors, a differential expression analysis (DGE) was performed revealing a large number of differentially expressed genes (Fig. 4H). These were then used to perform a Gene Set Enrichment Analysis on the forebrain lesions which highlighted pathways related to neuron differentiation, cell differentiation, and generally early developmental pathways, further supporting the undifferentiated nature and neural progenitor origin of these lesions (Fig. 4I).

### Stepwise differentiation and tumor development process is visible in transcriptomic data

**Fig. 5:**
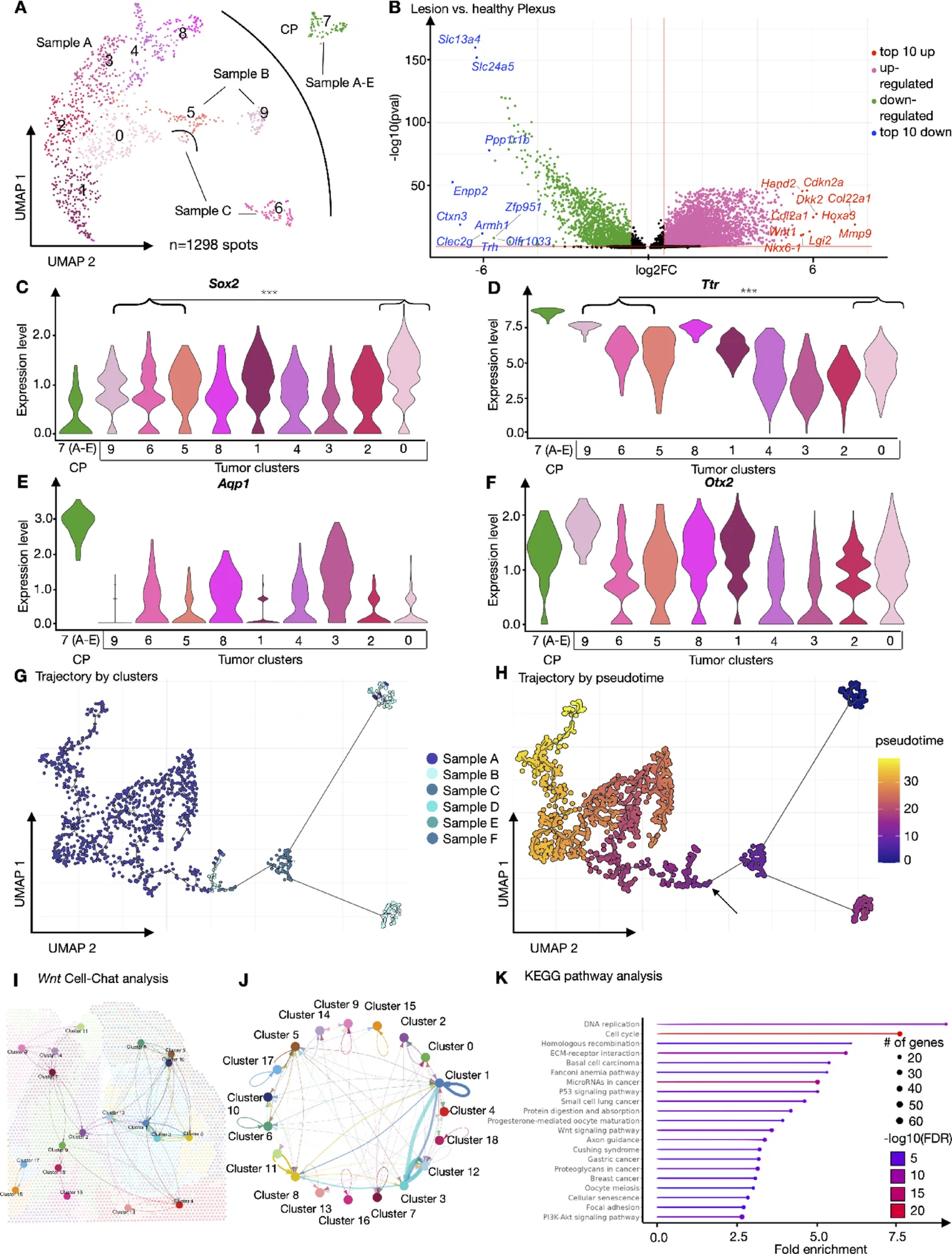
Heterogeneity in CPTs allows insight into tumor development and progression. **A** Reclustered UMAP consisting of healthy CP tissue and hindbrain tumor samples, numbered by Seurat clusters, different samples contributing different clusters, all healthy CP tissue is integrated together in cluster 7 (green). **B** Volcano plot showing differentially expressed genes between plexus tumors and healthy plexus, top10 upregulated genes annotated in red and downregulated in blue, DEG upregulated in tumors pink, downregulated green, cut-off values for significant DEG *p* < 0.05 and log2FC > 0.6. **C**–**F** Violin plots depicting different expression of *Sox2*, *Ttr*, *Aqp1,* and *Otx2* in various tumor clusters showing intratumoral heterogeneity. Sox2 expression was significantly lower in smaller lesions (clusters 9, 6, 5) compared to cluster 0 (FDR-corrected Wilcoxon rank-sum test, *p* < 0.0001). **G** Trajectory analysis colored by tumor sample showing different contributing samples to developmental time points, arrows mark further progressed spots of Sample B (light blue) and Sample C (darker blue). Mixed cluster of healthy plexuses was selected as root node. **H** Trajectory analysis colored by pseudotime showing tumor development. **I** Spatial network plot with *Wnt* signaling interaction based on Cell Chat for a representative sample (A in Fig. 4), tumor clusters are 1, 3 and 8, detailed analysis is available in Supplemental Fig. 8. The interaction strength of *Wnt* signaling is shown in the circle plot J), the dot colours indicate different Seurat clusters. **K** KEGG pathway analysis based on top200 DEG, circle size indicates number of genes in pathway, x-axis marks fold-enrichment, and color gradient denotes false discovery rate (FDR)

To gain further understanding of the CPC tumors and the observed tumor progression and developmental processes the spots were subsetted and reclustered (Fig. 5A) resulting in different subclusters from the tumor samples but a complete integration of healthy CP tissue from all samples. We then conducted a DGE (Fig. 5B) revealing downregulated genes such as *Slc13a4*, a transmembrane transport regulator, and *Slc24a5*, responsible for potassium dependent transport, associated with normal CP function and upregulated genes for example *Cdkn2a* and *Hand2* associated with tumorigenesis or epithelial-mesenchymal transition. Other upregulated genes like *Dkk2*, *Wnt1,* and *Hoxa3* are implicated in Wnt signaling, strengthening the link between tumor formation and signaling important for embryological plexus development. In transcriptome analysis of human tumor samples [19], Slc family genes also make up 122 of all 5045 significantly differently expressed genes. This is reproducible in our mouse tumors with large aberrant Slc expression (Fig. S5). *Col22a1* and *Col2a1* are in the top10 upregulated genes as well, and collagens have only recently been characterized as a key component of cell-to-cell interaction and significant tumor interaction networks in human pedB tumor samples [19].

Next, we analyzed the histologically observed stepwise dedifferentiation on a transcriptomic level and observed a concurring picture. Tumor expression of *Sox2*, *Aqp1*, *Ttr,* and *Otx2* is clearly different from healthy CP profile (Fig. 5C–F, green) but heterogenous within the tumor clusters (pink clusters). The smaller lesions (clusters 9, 6, and 5) have significantly less *Sox2* expression in comparison to cluster 0 (Fig. 5C) and retain more *Ttr* expression (Fig. 5D). In the bigger tumor sample, A, a heterogenous expression can be observed, suggesting a continuous tumor evolution during growth (see also Fig. S6).

We validated this using trajectory analysis with monocle3. The root node is made up of healthy tissue from all CP samples (Fig. 5G), and the tumor development can be traced throughout the clusters, the smaller lesions corresponding to earlier pseudotime (Fig. 5H) but also include some spots further along in tumor development (arrows).

Since ciliation is an essential feature of normal CP function and changes in ciliary structure consistently characterized our lesion, we examined ciliary developmental genes described in literature [11, 12] (Fig. S7) and observed an upregulation in embryological signaling in early tumor clusters (clusters 6, 9, 5, and 8). The temporal and spatially distinct plexus development was also visible on a transcriptomic level with the tumor consistently overexpressing caudal differentiating genes concurrent with the histologically observed anatomical origin (Fig. S7). These genes are significantly higher expressed in embryonal origin cells of the CP that contribute to the caudal part of CP tissue formation [12].

Within our spatially resolved data we analyzed receptor ligand interaction within the samples. Embryological pathways such as Wnt signaling where especially important for intratumoral signaling (Fig. 5I and J). Additional signaling pathways in embryological CP development, specifically BMP and Notch signaling were also active in intratumoral signaling (Fig. S8). When comparing the signaling state of healthy CP tissue and tumor clusters in our data, the importance of collagen for cell–cell interaction was demonstrated, adding to what can be seen in human data [19], as well as additional neurodevelopmental pathways such as the interaction of Pleiotrophin. The analysis also revealed *Igf2* as an intratumoral growth signal (Fig. S8).

No significant immune signatures were found in spot deconvolution of the samples and there was little immune marker expression in the overall dataset. This is consistent with rare immunogenic infiltration of the human CPC tumors (Fig. S9).

We finally performed a Kyoto encyclopedia of Genes and Genomes (KEGG) pathway analysis which revealed relevant upregulation of signaling associated with cancer or other specific entities (Fig. 5J). The enriched pathways show a strong overlap with KEGG analysis in human samples (Fig. S10). Both Wnt signaling as well as Pi3K-Akt signaling pathways enriched here could act as potential targets for therapeutic options. The overactivation of GLI2 in our model leads to an enriched Hedgehog signaling pathway which is not seen in the human data, even though the aberrant ciliogenesis in human tumors as well as previous analysis of human samples have indicated changes in hedgehog signaling as part of human tumor development [32].

### Tumor derived cultivated cells are growing in vitro and are sensitive to ATR and CDK inhibitors

**Fig. 6:**
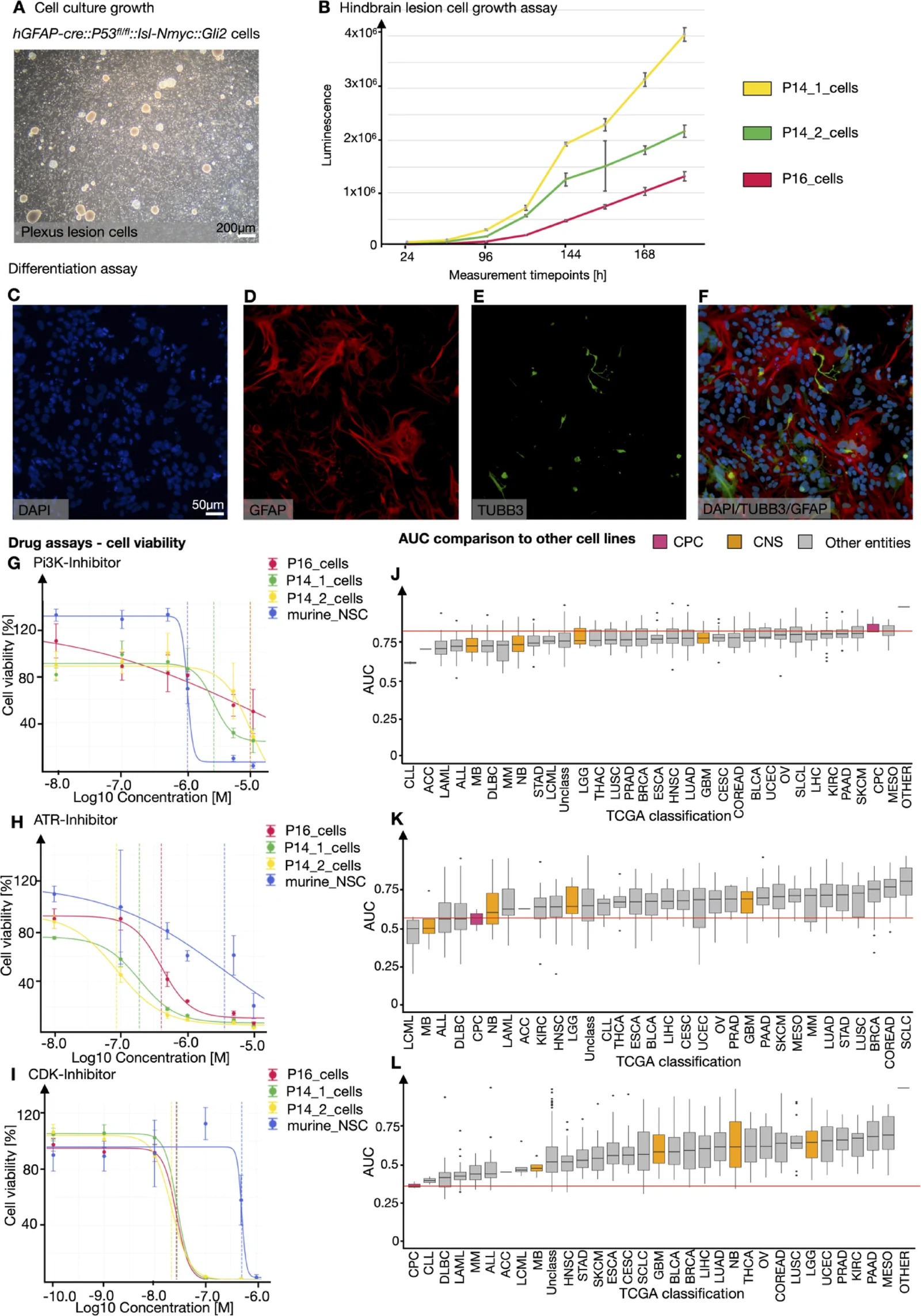
In vitro cultivated cells show vulnerability to CDK and ATR Inhibitors. **A** Hindbrain lesion cells grow in spheres in NSC medium. **B** Luminescence viability assays show consistent cell growth in n = 3 samples of different mice (2xP14, 1xP16) at 8 timepoints measured every 24 h. **C**–**F** Differentiation assay depicting DAPI stained cells in **C**, GFAP stained astrocytes in **D** and TUBB3 stained neuronal differentiated cells in **E**, merged image in **F**. Experiments were repeated with at least 3 independent images, TxRed positive area/cell and number of neurons/cell were calculated and did not differ significantly between samples. **G**–**I** Representative experimental dose–response curves of three different tumor cell lines and one mouse NSC control cell line with three replicates each, y-axis showing percent of viable cells, x-axis showing inhibitor concentration in M, error bars showing standard error of mean. Dashed lines correspond to IC50 values, pooled CPC IC50 means are significantly lower than NSC control IC50 values between CDK (*p* < 0.001, *t*-test) and ATR inhibitor (*p* < 0.05, *t*-test) treatments. **J**–**L** AUC comparison of different cells lines with integrated experimental data of four runs, y-axis AUC in range 0–1, x-axis different entities, pink = CPC tumor AUC, orange = CNS tumors. The red horizontal lines in **J**–**L** indicate the median AUC of the CPC cell line cohort to serve as a comparative baseline against other TCGA classifications

In order to use our model for potential therapeutic testing we cultivated tumor cells in NSC medium and observed spherical growth (Fig. 6A). The cells were continuously highly proliferative in luminescence growth assays (Fig. 6B), survived cell culture well, and we were able to cultivate them repeatedly, making them an easily usable tool for future experiments. They kept their marker expression and monociliary nature consistent with prior histological findings.

In a differentiation assay (Fig. 6C–F), the tumor cells showed the potential to diverge into major cell lineages such as astrocytes (Fig. 6E), neuronal (Fig. 6F) or oligodendrocytic cells.

To select potential drug testing candidates, we combined our molecular data with the previously available knowledge in both human CPC samples and other mouse model studies. The only therapeutic data available from humans is a cell line-based screening that identified CDK inhibitors, Pi3K-inhibitors and ATR-inhibitors among the top hits [39]. A large molecular analysis of 20 CPC also revealed PI3K-Akt pathways as highly associated with CPC cases [74]. In the KEGG pathway analysis the PI3K-Akt signaling pathway was a top result in both human and our transcriptomic data set (Fig. S10). The dedifferentiation process imposes replication stress which is evidenced by the importance of DNA repair pathways in our data. The top signaling pathway dysregulated in our mouse samples (Fig. 5E) is DNA metabolism. That is also relevant in human samples due to the large chromosomal instability reported [18, 19, 39]. That is targetable through CDK inhibitors for example Dinaciclib [4] a CDK 1, 2, 5, and 9 inhibitor that has previously been identified as a promising candidate in high-throughput drug screenings of another published mouse model [14]. ATR inhibitors [72] similarly take advantage of DNA replication stress mechanisms, and were successfully included in an in vivo treatment experiment by Martin et al. [39]. The PI3K inhibitor BKM120 [76] has good blood–brain-barrier penetration and was previously successfully tested in medulloblastoma and glioblastoma cell lines. Overexpression of relevant drug targets could also be seen in our transcriptomic data (Fig. S11). We therefore chose to test BKM120, Tuvusertib and Dinaciclib due to the evidence of DNA metabolism dependency available from both human data and our own transcriptomic data. All three drugs showed a growth inhibiting effect on the tumor cells (Fig. 6G–I) tested through Cell Titer Glo viability assays but only ATR and CDK inhibition exhibited a higher efficacy in tumor cells than in untransformed mouse NCS cells. In order to compare the results to previously available data and evaluate the specificity of therapeutic success, we employed the AUC in available data bases from a wide variety of cranial and extracranial tumor cell lines (Fig. 6J–L) [71]. Because no data were available for Tuvusertib we utilized data from a comparable ATR-inhibitor AZ-20. Here, both ATR inhibition and especially CDK inhibition show a lower AUC than most other cell lines and thus a higher targeting efficacy.

## Discussion

In this study we establish a new, early-onset mouse model to illuminate CPC progression processes. We demonstrate that hGFAP-driven expression of MYCN and Gli2, combined with p53 loss, induces CPTs in the fourth ventricle, in contrast to other combinations of these genes. The *hGFAP-cre::lsl-MYCN*^*fl/fl*^ results in a hypoplastic cerebellum [46], the p53 conditional knockout mice serve as a glioblastoma model [34] and the combined *hGFAP-cre::Tp53*^*fl/fl*^*::lsl-MYCN* mice develop large forebrain tumors resembling pediatric high grade glioma MYCN [56]. Our mouse model shows a strong recapitulation of human tumor features. The arising lesions bear a close histological resemblance to human CPC tumors, fulfilling WHO criteria and exhibiting similar architecture and marker expression. A key difference between mouse and human CPCs is their anatomical location. Our CPCs are confined to the fourth ventricle, while human CPCs primarily occur in the lateral ventricle [69]. This difference may result from the expression pattern of the *hGFAP* promoter, which excludes the lateral CP. SHH signaling has previously been implicated to be especially relevant in embryological 4th ventricle plexus development in mice but further research on humans and this pathway’s interplay and effect on CP development is still needed. The formation of tumors based on SHH pathway alterations in combination with *Tp53* loss, the so far only reliably recurring mutation in human CPCs, highlights the network’s role in plexus tumor development. Our mice exhibit higher tumor penetrance and earlier onset (P18 compared to P200, P160, or P150) compared to any of the previous models, while still being viable [14, 66, 68]. This parallels the clinical reality of human CPCs, which predominantly affect very young children. The aggressive growth and metastasis patterns also aligns with human data [35, 60, 69, 73].

Focusing on the processes leading to tumor initiation and progression we observed and characterized tumor development beginning with small lesions as early as P5, which we identified by chromatin and nuclear changes. We show stepwise dedifferentiation and changed marker expression starting with *Sox2* upregulation and *Ttr* loss. Then, *Aqp1* and finally *Otx2* expression is lost. Concurrent changes in ciliary structure further support the relevance of SHH signaling. Monociliary cells occur both in our model and in human CPCs which suggests a malignant reversion to embryonic signaling states in both species. Our spatial trajectory analysis refines this understanding by identifying a developmental axis were tumor cells re-engage specific embryological programs. The embryological CP development is well characterized in mice but needs further research in humans.

We generated the first spatially resolved transcriptomic molecular dataset for a mouse CPC model. The tumors were identified as most closely matched to CP transcriptional profiles but clearly displayed gene expression differences. We expanded understanding of dedifferentiation patterns and tumor formation at the transcriptomic level. The observed heterogeneity in tumor clusters allowed us to track tumor development and observe a pattern of healthy, mature CP tissue transitioning to a state that resembles embryological CP before further tumor progression leads to a loss of CP-specific signature and function. The signaling interaction analysis reveals that this dedifferentiation is supported by a remodeled signaling niche. We identified a robust upregulation of Wnt, BMP, and Notch signaling networks, which are spatially organized to maintain tumor proliferation. Furthermore, the extensive collagen interaction network observed in our model aligns with recent findings in human pedB tumors, pointing to a critical role for the extracellular matrix (ECM) in CPC progression. We showed parallels with human transcriptomic analyses, as many differentially regulated genes in mice also showed significant expression changes in human data, and the transcriptomic signatures map closely together in a wide cross-species analysis including multiple different tumor types. This allows insight into the development of these tumors and underscores the value of model systems for understanding tumor progression. Additionally, Slc family genes were dysregulated in both mouse and human CPCs. Hydrocephalus in mice and human tumors might be aided through Slc dysregulation as well as physical blockade of CSF flow through tumor formation [19].

Although *TP53* mutations are common in CPCs, no other recurring driver mutations have been identified in humans. Human tumors exhibit broad genomic instability, characterized by recurring copy number variations and chromosomal gains and losses. We did not observe these in the molecular data of our mouse model (Fig. S12). The relevance of copy number variations for tumor initiation was previously modeled successfully and used to identify potential oncogenes *Taf12, Nfyc,* and *RAD54L,* which were interpreted as addiction to DNA-repair and epigenome remodeling capacities [66]. These characteristics are missing here likely due to the sufficiency of the tumor drivers as initiators of an oncogenic expression profile rather than a more general chromosomal instability. The transcriptomic profile of our model and previously published ones still share important similarities (Fig. S13), and other parameters such as the onset, tumor development, growth aggressiveness, and histology indicate a strong resemblance to human tumors. Our approach thus allows for the tracking of tumor progression. The role and pattern of copy number variations remain an important question for future research.

Cells derived from our mouse model were successfully cultivated in vitro. Given the rarity of these neoplasms and the scarcity of human cell lines, these mouse-derived lines offer crucial opportunities as a research tool. Nonetheless, the differences, particularly the absence of copy number variations, might lead to different responses to proposed treatment regimens. The evident relevance of DNA metabolism in human data and our mouse model prompted preliminary testing of three targeted substances.

Both ATR and CDK inhibition demonstrated strong cell-killing efficacy and a potential for new treatment options. Because such in vitro data are not sufficient, further testing is needed to confirm these effects and compare them with standard treatments, including radiation and chemotherapy. This presents continuous challenges due to the short window to apply therapeutics as well as the complex chemotherapeutic treatment regimen used in patients. The concomitant forebrain lesions also warrant further consideration. Given their transcriptomic resemblance to SCZ neural progenitors, future studies employing SCZ-specific lineage tracing or single-cell multi-omics could definitively establish their exact cell of origin and molecular evolution. Importantly, the presence of these secondary lesions does not diminish the biological validity or interpretation of the hindbrain CPC model, as the two tumor entities are anatomically and transcriptomically distinct. However, the concurrent growth of these forebrain tumors could act as a confounding factor for in vivo survival analyses. This limitation can be effectively mitigated by utilizing our established primary CPC cell lines for orthotopic transplantation into syngeneic hosts, as previously described, for example, with Medulloblastoma cells [16], providing an isolated and reliable system for preclinical drug screening. The CP is a complex, often overlooked tissue critical for nervous system development and maintenance. While genetically engineered mouse models have inherent limitations in mimicking human diseases, they enable detailed tracking of tumor progression, and since CPC tumors remain rare and poorly understood, we believe our mouse model provides valuable insights into their development and biology.

## Supplementary Information

Additional file1 (PDF 30570 kb)

Additional file2 (XLS 19 kb)

Additional file3 (XLS 303 kb)

Additional file4 (XLS 21 kb)

Additional file5 (XLSX 16 kb)

## Data Availability

All transcriptomic data are available under the GEO Accession number [GSE289052](https:/www.ncbi.nlm.nih.gov/geo/query/acc.cgi?&acc=GSE289052).
