# Supplementary material for: Overactivation of SHH signaling induces a cascade of dedifferentiation and oncogenesis in a new mouse model for choroid plexus carcinoma
Source: Acta Neuropathol Commun. 2026 Aug 21;14:176. doi: 10.1186/s40478-026-02402-y (PMC13499314; doi:10.1186/s40478-026-02402-y)
Supplement: Supplementary file 1 [file 40478_2026_2402_MOESM1_ESM.pdf]

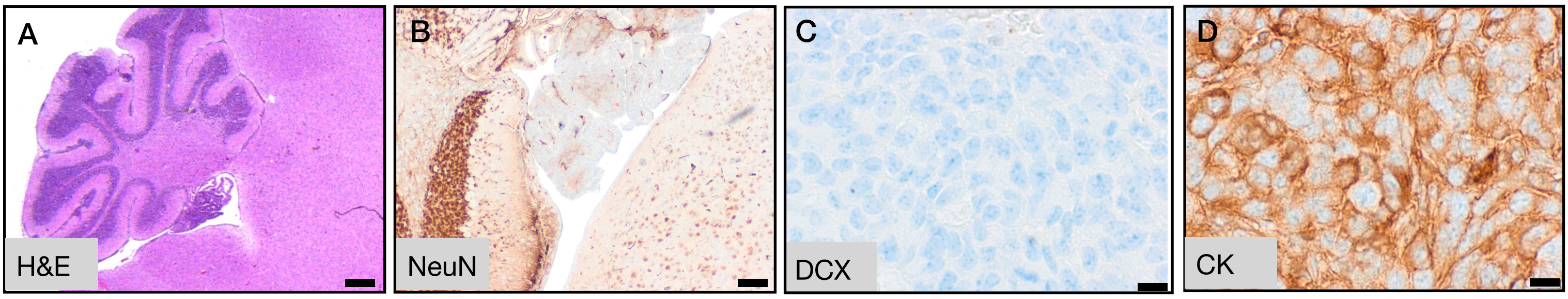

Supplementary Figure 1: Additional histological characterisation
Additional characterisation of murine CPC sample, showing NeuN negativity **(B)** and Doublecortin **(C)** negativity. This highlights the difference of our CPC model to SHH medulloblastoma models. Positive expression of Cytokeratin **(D)** similar to human tumors. Scale bar A ≙ 200 µm, scale bar B ≙ 100 µm, scale bar C, D ≙ 50 µm

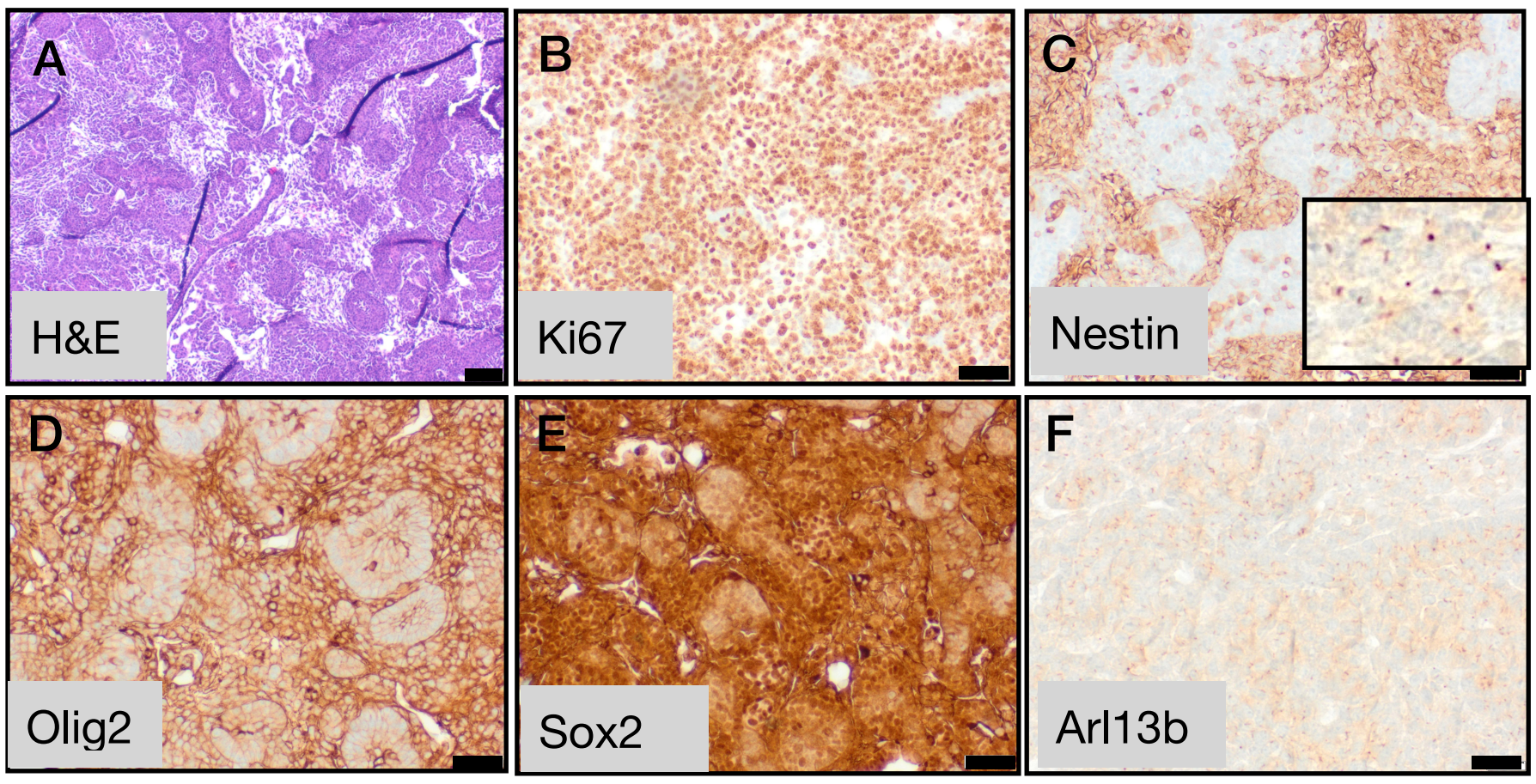

Supplementary Figure 2: Trigeminal metastasis
Trigeminal metastasis in H&E stain (**A**) showing high proliferation (**B**), Nestin expression (**C**), Olig 2 negative nuclei (**D**), and Sox 2 positive nuclei (**E**). Arl13b stain shows monocilliated cells (**F**). Scale bar A ≙ 100 µm, scale bar B-F ≙ 50 µm

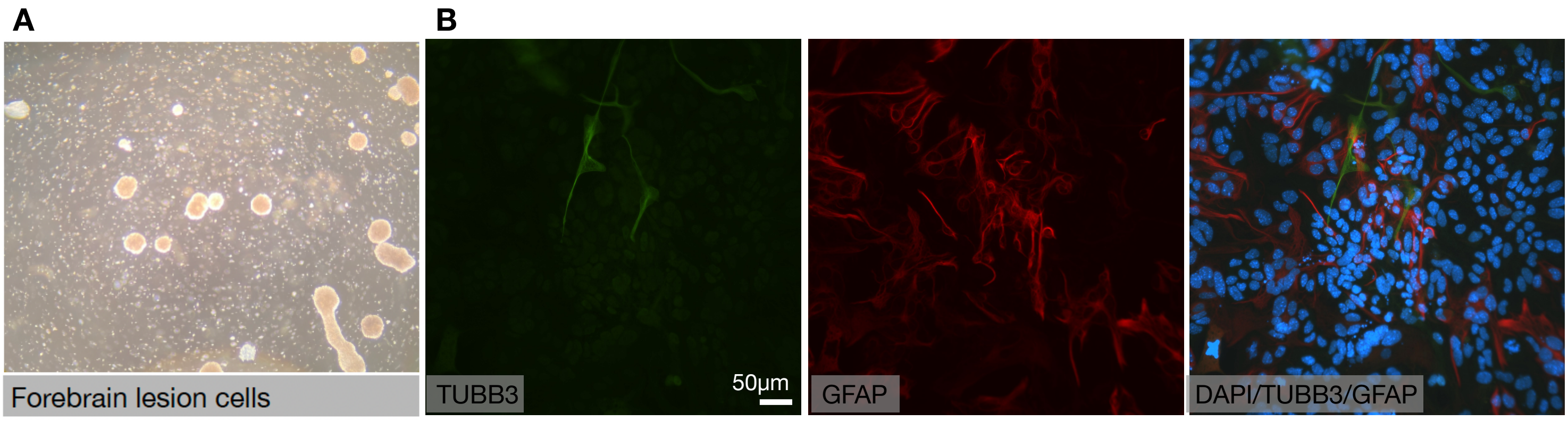

Supplementary Figure 3: Cell culture growth of forebrain lesions
Forebrain lesion cells show neurospheric growth in cell culture (**A**) and a broad ability to differentiate (**B**) into different lineages based on a similar differentiation assay and methodology as in Fig. 6 C-F

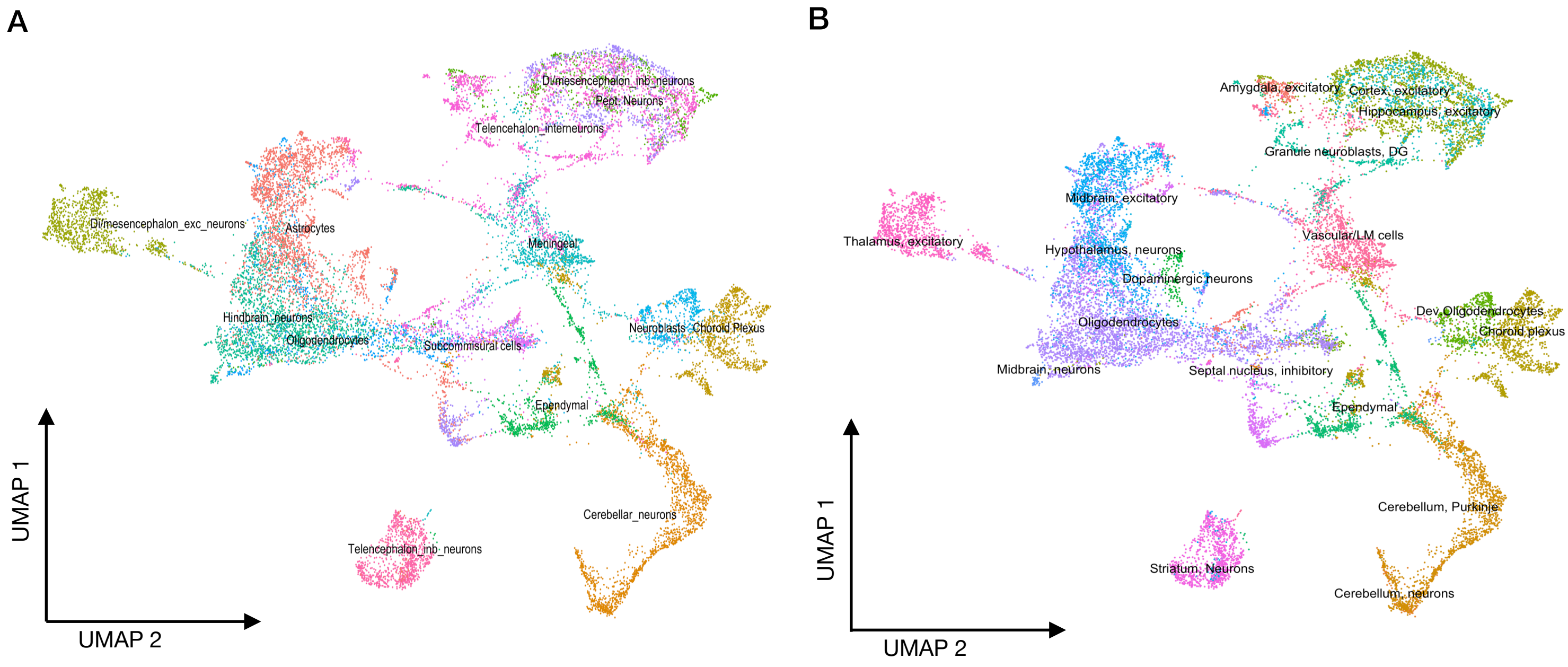

Supplementary Figure 4: Annotation based on different references
UMAP based on Fig. 4G, referencing atlas Zeisel et. al. 2018 in **A**) and Shi et. al. 2023 in **B**) confirming confident mapping and classification of spatial data using scType.

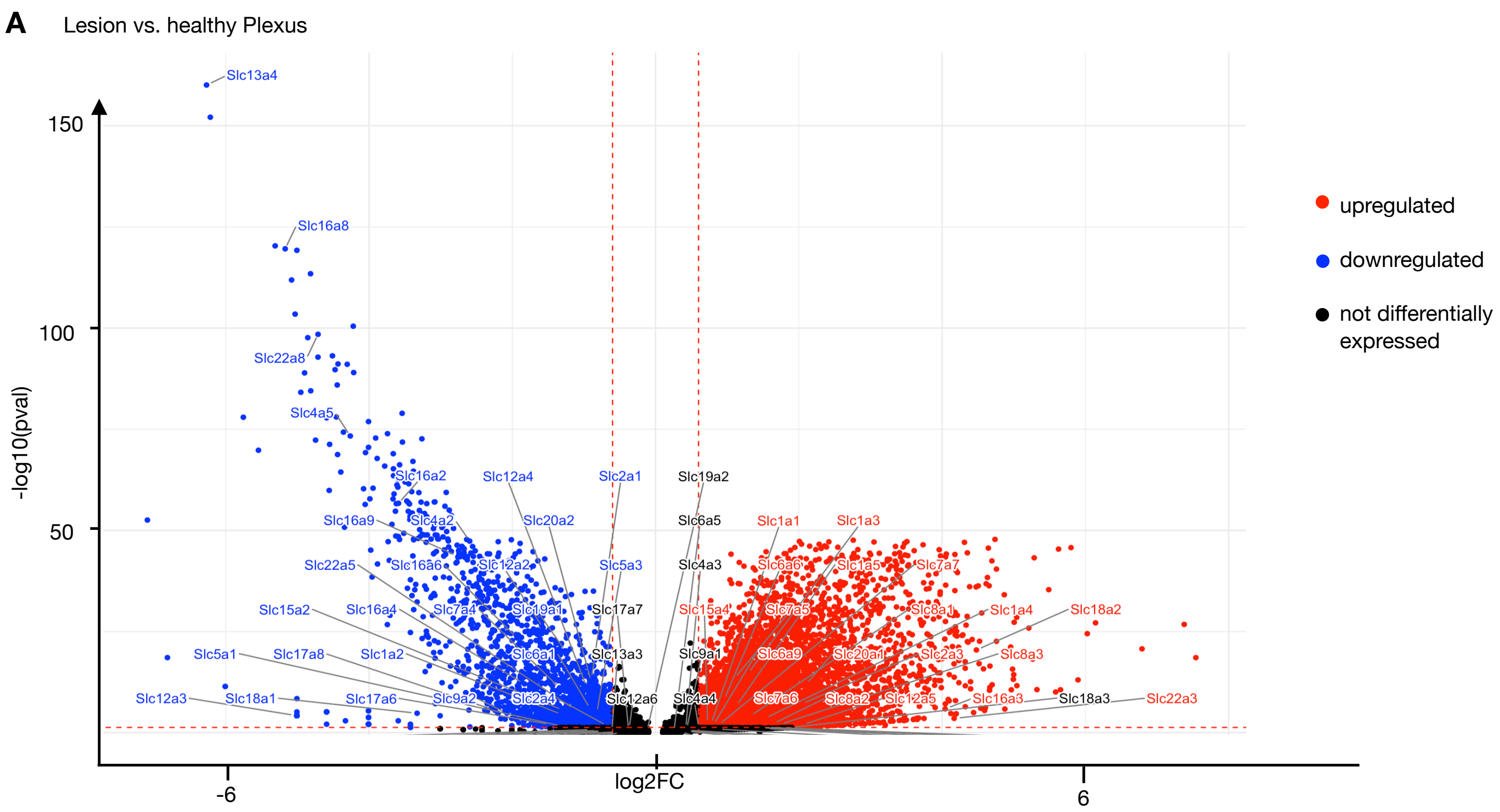

Supplementary Figure 5: SLC family genes
Volcano plot showing DEG between tumor clusters and healthy plexus, all SLC family genes are annotated, genes written in red are upregulated, genes written in blue are downregulated

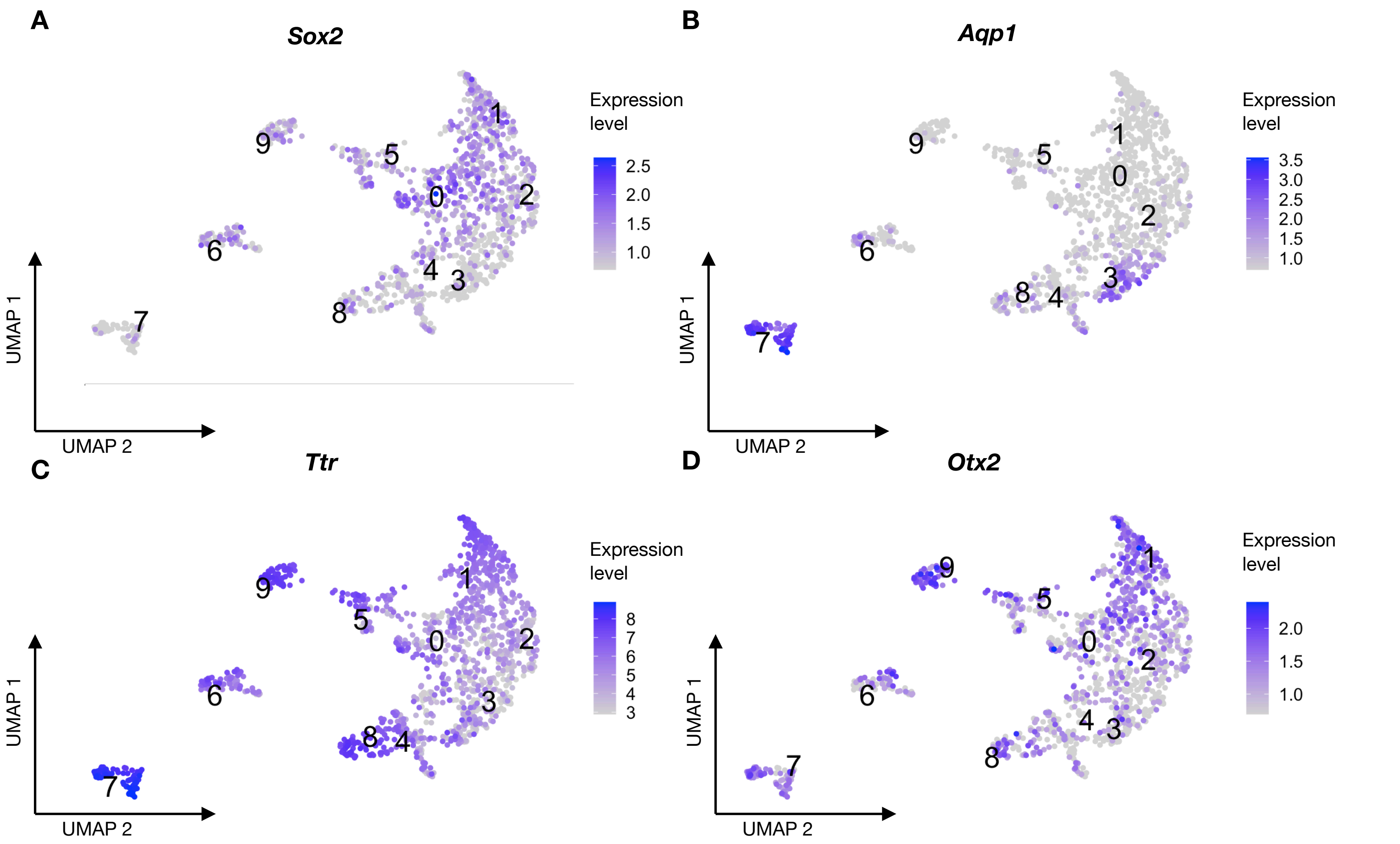

Supplementary Figure 6: Heterogeneity in marker expression throughout tumor development
UMAPs showing the difference in marker expression based on separate tumor clusters, as seen in Fig. 5A). *Sox2* (**A**), *Aqp1* (**B**), *Ttr* (**C**) and *Otx2* (**D**) are shown; the colour corresponds to the expression level.

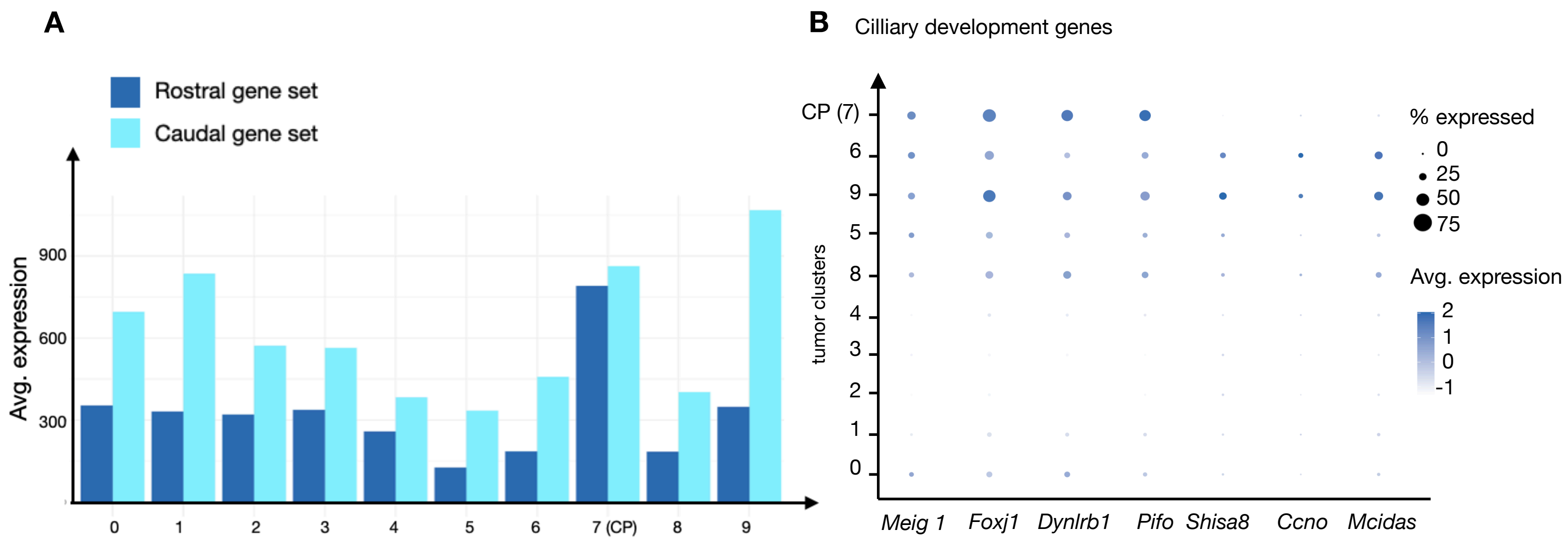

Supplemental Figure 7: Rostral/Caudal differentiating gene sets
A) Bar plot showing average expression based on clusters from Fig.5. Rostral and caudal gene sets from Dani et. al. 2021 are used, healthy CP tissue in cluster 7 shows almost equal expression of rostral and caudal gene sets, all other tumor clusters show a higher expression for caudal genes. **B**) Ciliary developmental genes from Dani et.al. reference data, color gradient denotes expression level, size of circles represents the percent of spots expressing the gene, earlier tumor clusters show higher expression of ciliary developmental signature

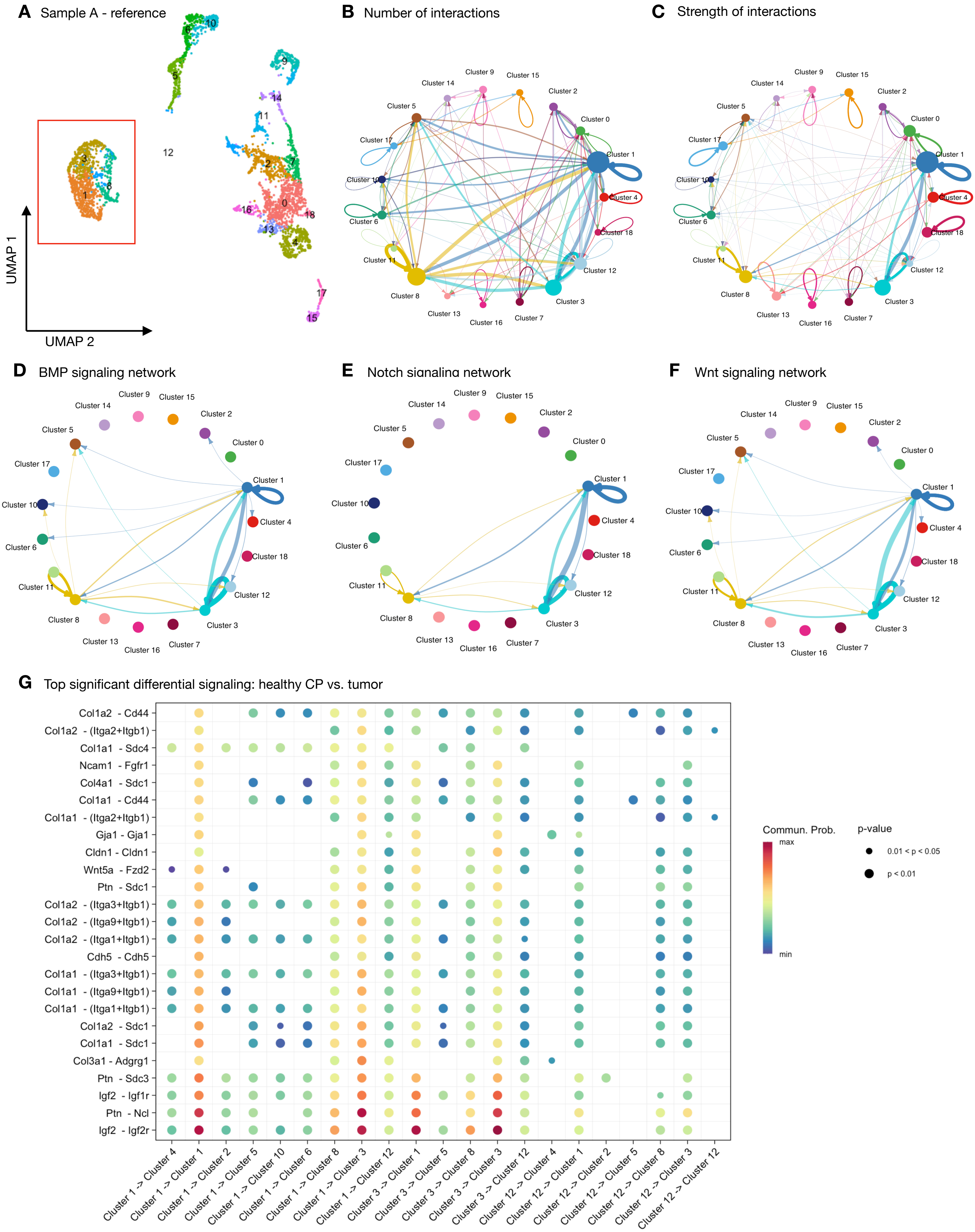

Supplemental Figure 8: Signaling pathways in Cell-Cell Communication

A) UMAP showing a representative sample (A - as seen in Fig. 4), red box marks tumor clusters 1, 3 and 8. For Cell-cell interactions between all clusters in A) the dot size is proportional to the number of inferred links (both outgoing and incoming) associated with each group in B) and strength in C), the dot colours indicate different groups. D) - F) circle plots of embryological developmental pathways BMP signaling, Notch signaling and WNT signaling G) Dot plot with top significantly expressed signaling pathways in tumor clusters and healthy CP tissue, color gradient denotes communication probability

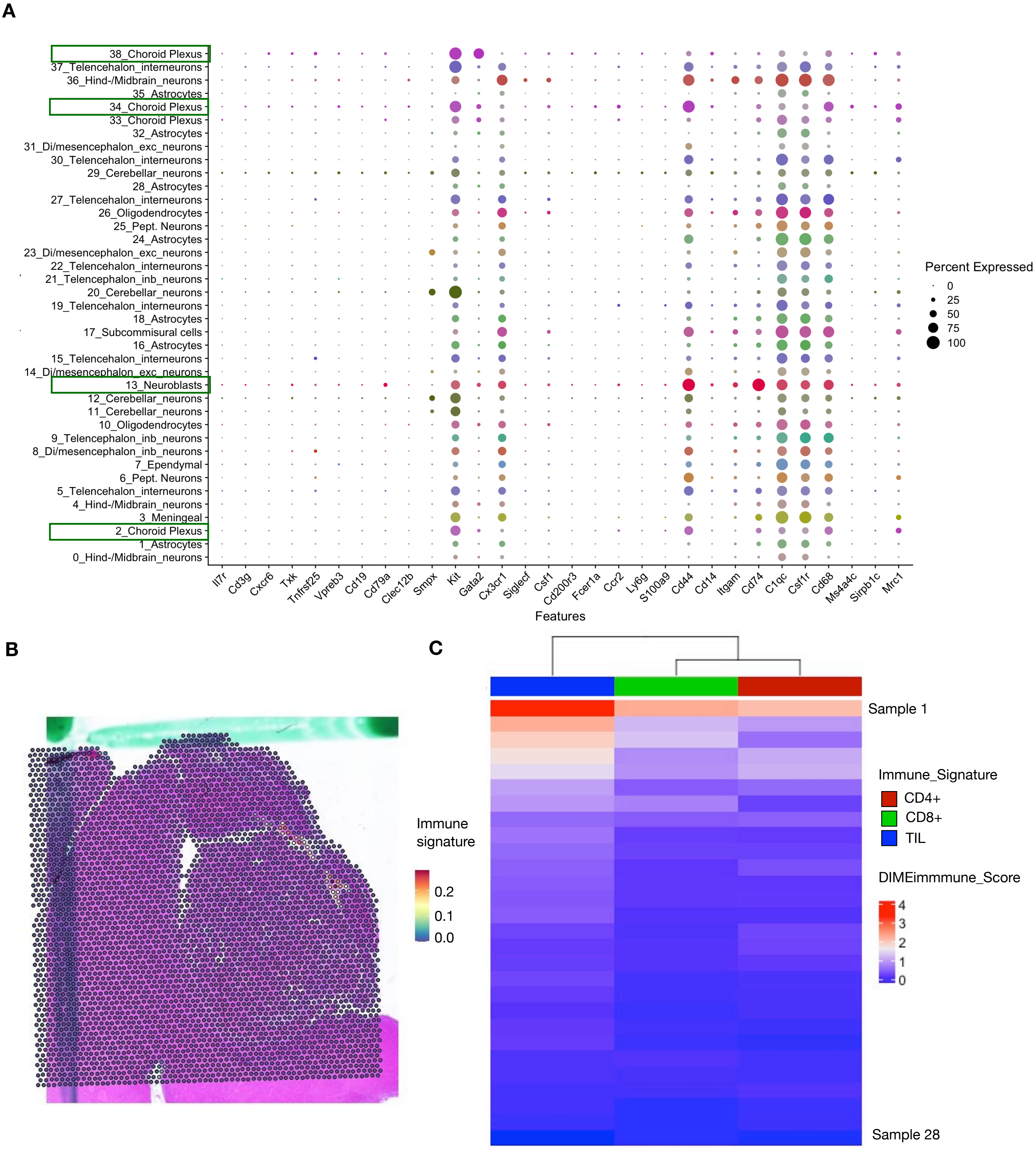

Supplemental Figure 9: Immune infiltration

A) Immune marker genes in clusters based on Fig. 4, showing no difference in immune signatures in any specific subset of the spatial transcriptomic data, green boxes highlighting clusters consisting tumor cells **B**) Exemplary spot deconvolution of one sample based on murine immune reference data showing little immune signature in tumor spots **C**) DIMEImmune analysis of human CPC methylation data differentiating between CD4+, CD8+ and Tumor infiltrating lymphocytes (TIL), the deconvolution demonstrates little immune cell portion in human tumors.

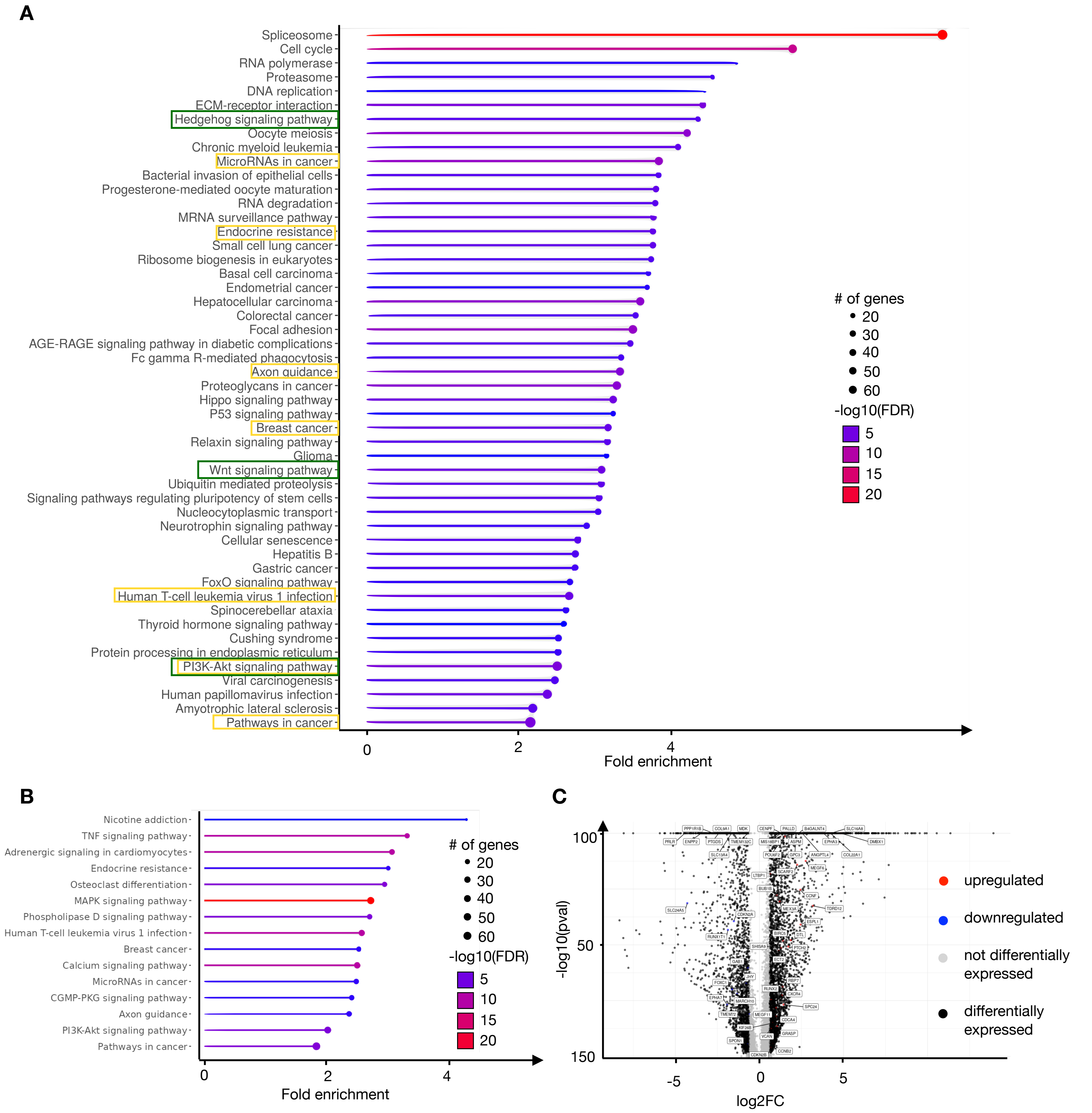

Supplemental Figure 10: DEG overlap

A) showing Top 50 KEGG Differential in mouse samples, all marked pathways in yellow are overlapping with the top10 pathways in human samples shown in **B**). Green highlights Shh and Wnt signaling as embryological signature pathways **C**) Volcano plot of all DEGs in human pedB CPC tumor samples vs health ChP, red and blue annotated genes are differentially expressed top 20 genes in mouse transcriptomic analysis, all mouse genes that are up/downregulated are also up/downregulated in human data

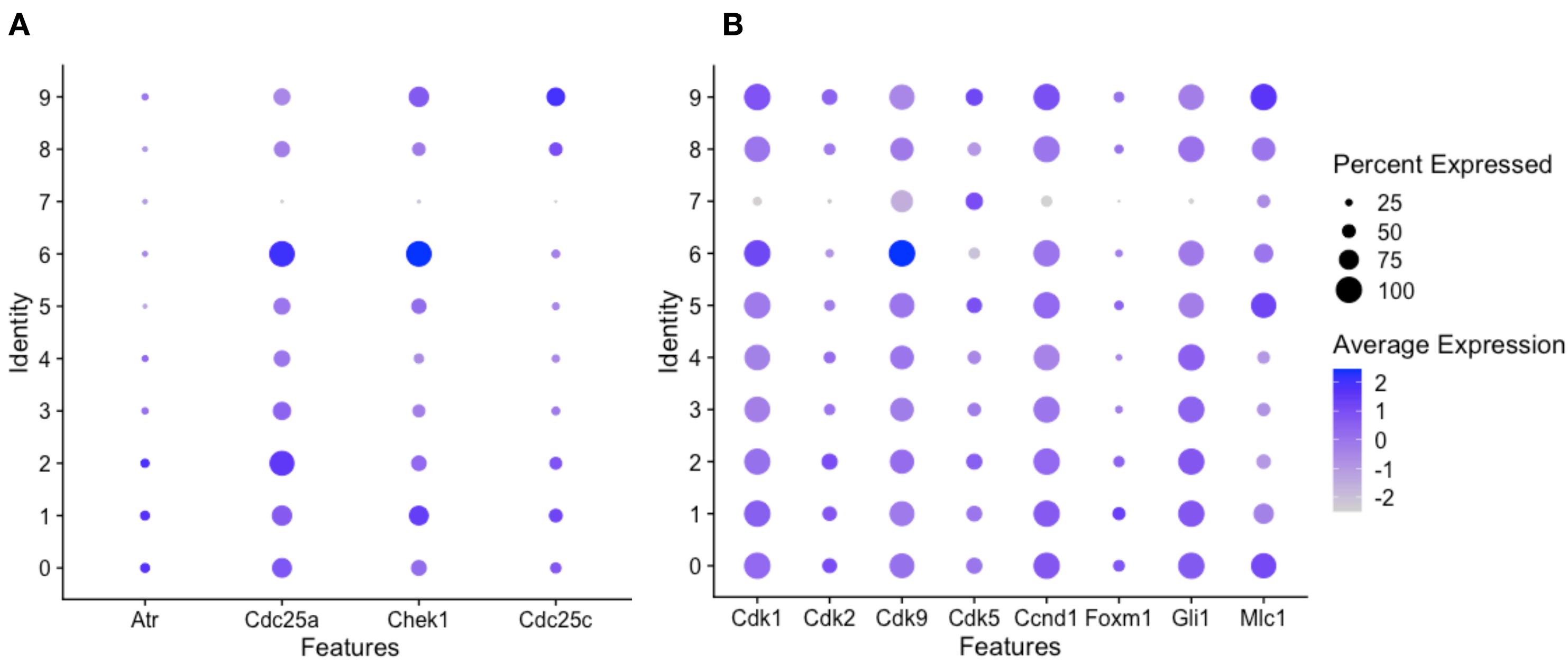

Supplemental Figure 11: CDK and ATR targets
Expression of ATR target genes (**A**) and CDK target genes (**B**) in tumor clusters and control plexus cluster 7, based on Fig. 5. Genes were selected based on literature search and DrugBank () available data. An over expression of most CDK and ATR targets is visible.

A

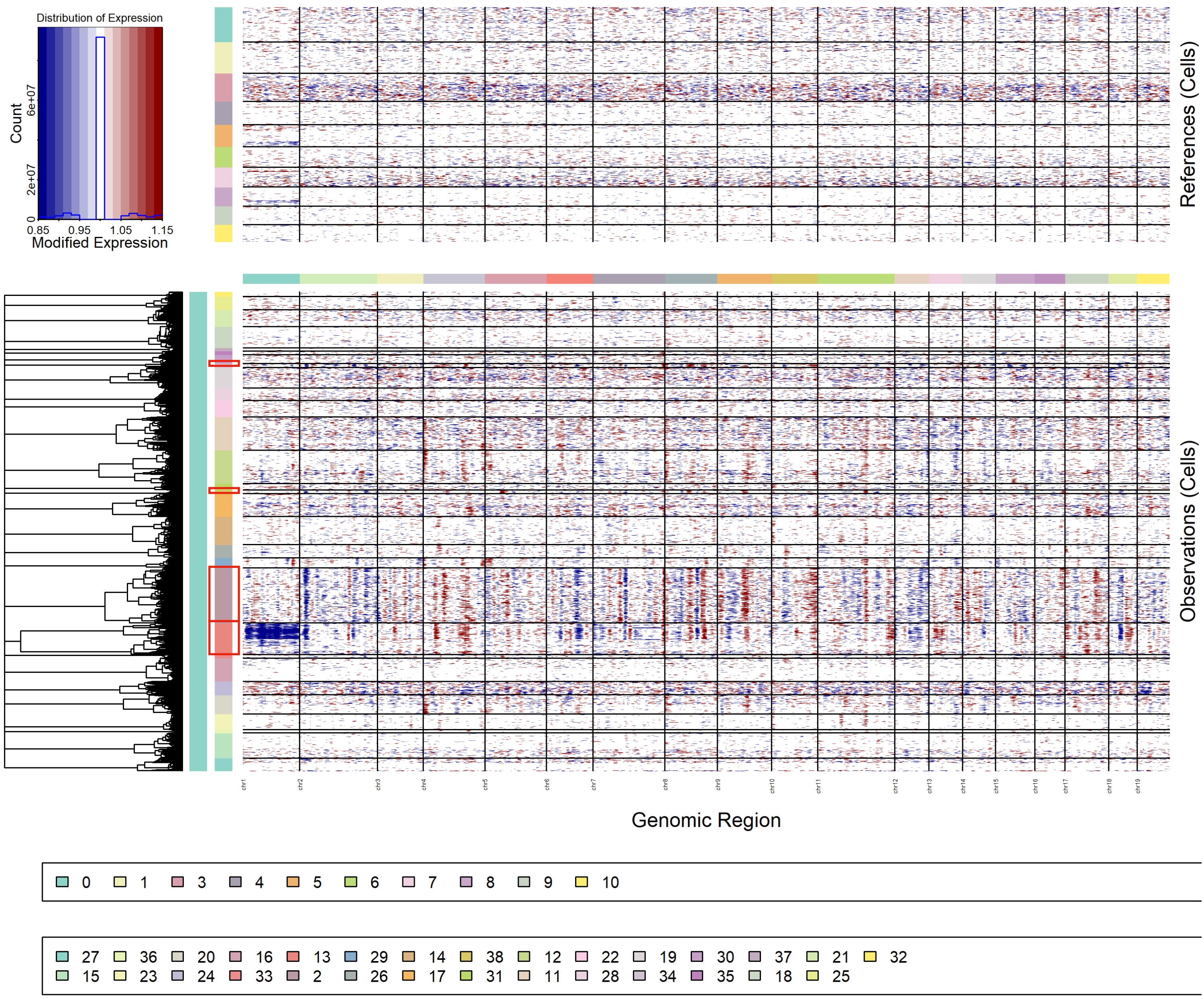

Supplemental Figure 12: Copy number changes

A) InferCNV analysis of all clusters based on Fig. 4 showing no large copy number variations or large scale chromosomal gains or losses as visible in human tumors. Red boxes mark the tumor cell clusters 2,13,34 and 38. Reference data are constructed from other clusters with expected flat copy number profile.

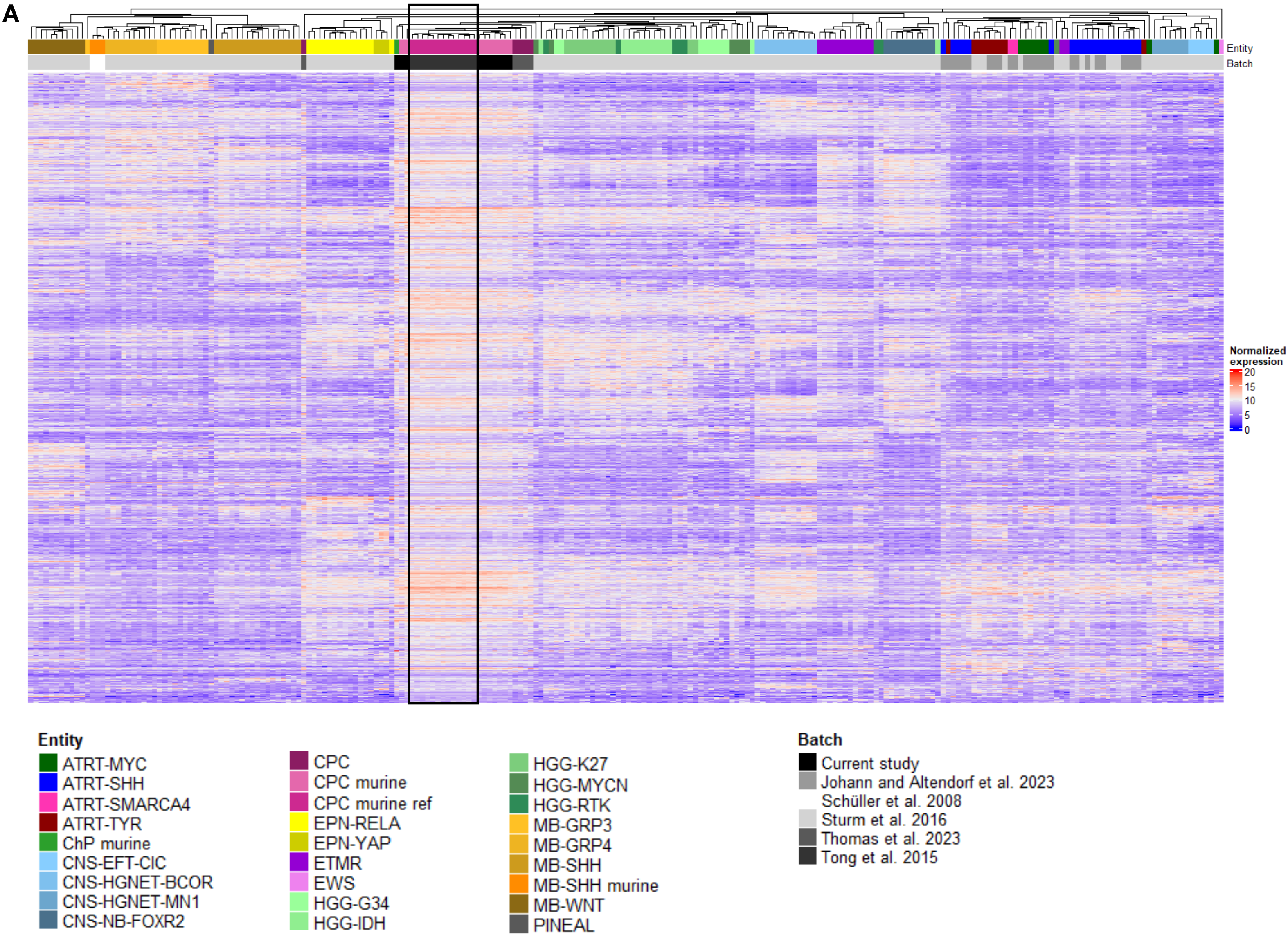

Supplemental Figure 13: Comparison to other CPC mouse models

A) Heatmap integrating the transcriptomic data available from Tong et. al. 2015 with the data of Fig. 4K. This demonstrates that both our model and the mouse CPC model from Tong et. al. have transcriptional similarities to human CPC samples. The top 1000 overlapping differentially expressed genes across al samples are used to construct the transcriptomic profile.
